# Comparative genomics of *Fusarium* species causing Fusarium ear rot of maize

**DOI:** 10.1371/journal.pone.0306144

**Published:** 2024-10-18

**Authors:** Owen Hudson, Colton D. Meinecke, Jeremy T. Brawner

**Affiliations:** 1 Department of Plant Pathology, University of Florida, Gainesville, FL, United States of America; 2 Warnell School of Forestry and Natural Resources, University of Georgia, Athens, GA, United States of America; 3 Genics Ltd, Queensland, Australia; Benemérita Universidad Autónoma de Puebla: Benemerita Universidad Autonoma de Puebla, MEXICO

## Abstract

Fusarium ear rot (FER), caused by the fungal pathogen *Fusarium verticillioides*, stands as one of the most economically burdensome and pervasive diseases affecting maize worldwide. Its impact on food security is particularly pronounced due to the production of fumonisins, toxic secondary metabolites that pose serious health risks, especially for livestock. FER disease severity is complex and polygenic, with few resistance (R) genes being identified for use in breeding resistant varieties. While FER is the subject of several breeding programs, only a few studies have investigated entire populations of *F*. *verticillioides* with corresponding virulence data to better understand and characterize the pathogenomics. Here, we sequenced and compared the genomes of 50 *Fusarium* isolates (43 *F*. *verticillioides* and 7 other *Fusarium* spp.) that were used to inoculate a diverse maize population. Our objectives were to elucidate the genome size and composition of *F*. *verticillioides*, explore the variable relationship between fumonisin production and visual disease severity, and shed light on the phylogenetic relationships among the isolates. Additionally, we conducted a comparative analysis of the nucleotide variants (SNPs) and the isolates’ effectoromes to uncover potential genetic determinants of pathogenicity. Our findings revealed several promising leads, notably the association of certain gene groups, such as pectate lyase, with disease severity. These genes should be investigated further as putative alleles for breeding resistant maize varieties. We suggest that, beyond validation of the alleles identified in this study, researchers validate each phenotypic dataset on an individual basis, particularly if considering fumonisin concentrations and when using diverse populations. Our study underscores the importance of genomic analysis in tackling FER and offers insights that could inform the development of resilient maize cultivars. By leveraging advances in genomics and incorporating pathogen populations into breeding programs, resistance to FER can be advanced.

## Introduction

Fungal species occupy a diverse range of ecological niches and display a variety of biological strategies from pathogenic to commensalistic to saprophytic. In addition to being cosmopolitan in distribution, pathogenic *Fusarium* (Nectriaceae, Hypocreales) species can infect a wide range of organisms including humans, other animals, plants, and microorganisms [1–3]. *Fusaria* also produce toxic secondary metabolites, such as trichothecenes, zearalenones, and fumonisins, which can contaminate foods or act as pathogenicity factors [4–6]. *Fusarium* has previously been described by its sexual morphs *Gibberella* and *Nectria*, however the unified taxonomy using the asexual epithet *Fusarium* is predominant and considered standard since the “one fungus, one name” nomenclatural revision [7–9]. The genus is currently divided into different species complexes, and within each complex, species still differ greatly in trophic modes and habitats [2,10]. Phenotypic and morphologic characterization is important, and sometimes necessary, for identification to species and beyond, as some *Fusarium* species have subspecies or races which are specific to particular plant species or groups. Observational characteristics, however, are often not sufficient to determine an individual isolate’s identity in detail, so genomic information is used to improve specificity [11–13].

*Fusarium verticillioides* (*Fv*, previously *F*. *moniliforme* Sheldon, teleomorph *Gibberella moniliformis* Wineland) is a nearly ubiquitous pathogen infecting maize (*Zea mays* L.). On maize, *Fv* acts as a hemibiotroph and an endophyte, living asymptomatically in the roots, stem, and ears, while able to cause rot diseases in all tissue types [14–16]. On maize ears, *Fv* is the primary cause of the disease Fusarium Ear Rot (FER), but other species can infect maize ears such as *F*. *proliferatum*, *F*. *subglutinans*, and *F*. *graminearum* (termed Gibberella ear rot in such cases) [17–19]. Yield losses from FER are not primarily driven by seedling death or stalk and kernel rot but are instead largely the result of mycotoxin contamination. *Fv* (and a few other species to a lesser degree) can produce fumonisins, a group of mycotoxins which cause much greater yield losses through the necessary disposal of contaminated grain. Consumption of fumonisin causes several deadly livestock disorders (e.g., leukoencephalomalacia and pulmonary edema), cancers in rodents, and is associated with neural tube defects and esophageal cancer in humans. While fumonisin has not been confirmed as a virulence factor, it is thought to be produced in response to tissue damage, pH changes, or other stresses on the host and the pathogen [20–23].

Individual *Fv* isolates differ greatly in their pathogenicity, disease severity, and ability to produce fumonisin [24,25]. Experimental knockouts of the *FUM* biosynthetic gene cluster indicate that a loss in fumonisin production does not result in reduced disease severity [16,26,27]. Variations in disease symptoms are also due to the multiple methods by which *Fv* invades and infects maize plants. Transmission is commonly inherited vertically, with most maize seed containing *Fv* within the kernels that are harvested and planted, providing the fungus with an advantage in developing as part of the new plant’s microbiome [28,29]. Pathogenesis typically occurs when the plant or fungus receives stress, or another environmental trigger induces a switch from biotrophy to necrotrophy [21]. Two other infective methods, through maize silks and into open wounds, are considered a more direct path to pathogenicity. *Fv* takes advantage of the cellular cracks or wounds that occur during the growth and development of the plant or when an insect damages an ear. The infective propagules of *Fv* (macroconidia and microconidia) can either invade through the wounds or coopt the silk and pollen tube to travel into the ear directly, implying that an intricate molecular relationship between host and pathogen has developed [30,31]. While genetic effects from both the host and the pathogen govern FER, disease severity is also influenced by environmental conditions, complicating the identification of specific disease-related genes [32,33].

In other comparative genomic studies on *Fusarium*, and for plant pathogens generally, focus has been placed on gene-for-gene (GFG) interactions, where interactions between pathogen effectors and the matching host resistance (*R*) genes regulate disease severity [34,35]. Identifying pathosystem-specific effectors and R gene interactions has been used to develop disease resistant plant varieties. When disease resistance is described as qualitative, it implies that one or few genes with large effects determine disease severity. Conversely, genetic parameters and molecular studies suggest that many genes contribute to pathogenicity and resistance in the FER pathosystem. This quantitative form of resistance makes detection of the genes regulating disease severity more difficult [36]. Fungal pathogens like *Fusarium* overcome resistance by evading or suppressing the host immune response, primarily through genetic diversification of pathogenicity genes such as effectors. Molecular studies have shown greater success detecting pathogenicity genes for qualitative diseases compared to quantitative diseases. However, advancements in sequencing, gene prediction, and functional annotations are improving our ability to detect genes regulating quantitative resistance [37–40]. Many *Fusarium* species can act as endophytes or mild pathogens on a large host range while still being virulent on a narrower host species/cultivar range [21,41–44]. These dynamics suggest that many of these pathosystems are quantitatively controlled while specific qualitative genes can define host specificity. Through various comparative studies, *Fusarium* species are known to share a large genomic core with additional accessory or lineage specific chromosomes that control host specificity and disease severity [45–48]. *Fv* is not known to have accessory or lineage specific chromosomes but there are discrepancies in chromosome number which opens the possibility of isolates containing additional genetic information [49]. As *Fv* and the FER pathosystem do not have many studies investigating the genetic dynamics of disease severity, a detailed genomic investigation may facilitate improvements in breeding resistant maize varieties that can be used to lower fumonisin contamination in the global food supply.

The objective of this research is to provide genomic data of 50 *Fusarium* isolates, evaluate the relationship between disease severity and groups of predicted genes/proteins commonly associated with FER severity, identify accessory sequences providing genetic diversity, and establish a basis for subsequent research on pathogenicity and resistance in the FER pathosystem. In this study, we present genome assemblies and comparisons among 50 *Fusarium* isolates, primarily *Fv*, and incorporate disease severity data that was gathered by inoculating maize ears with the 50 isolates over the course of three growing seasons. Analyses focus primarily on estimating the quality of genome assembly and alignment to reference genomes with genes predictions used to create phylogenetic trees from both core genes and putative effectors. To the best of our knowledge, this study compares the largest number of *Fusarium* genomes that have also been characterized for virulence by assessing the severity of FER symptoms.

## Results

**Fig 1:**
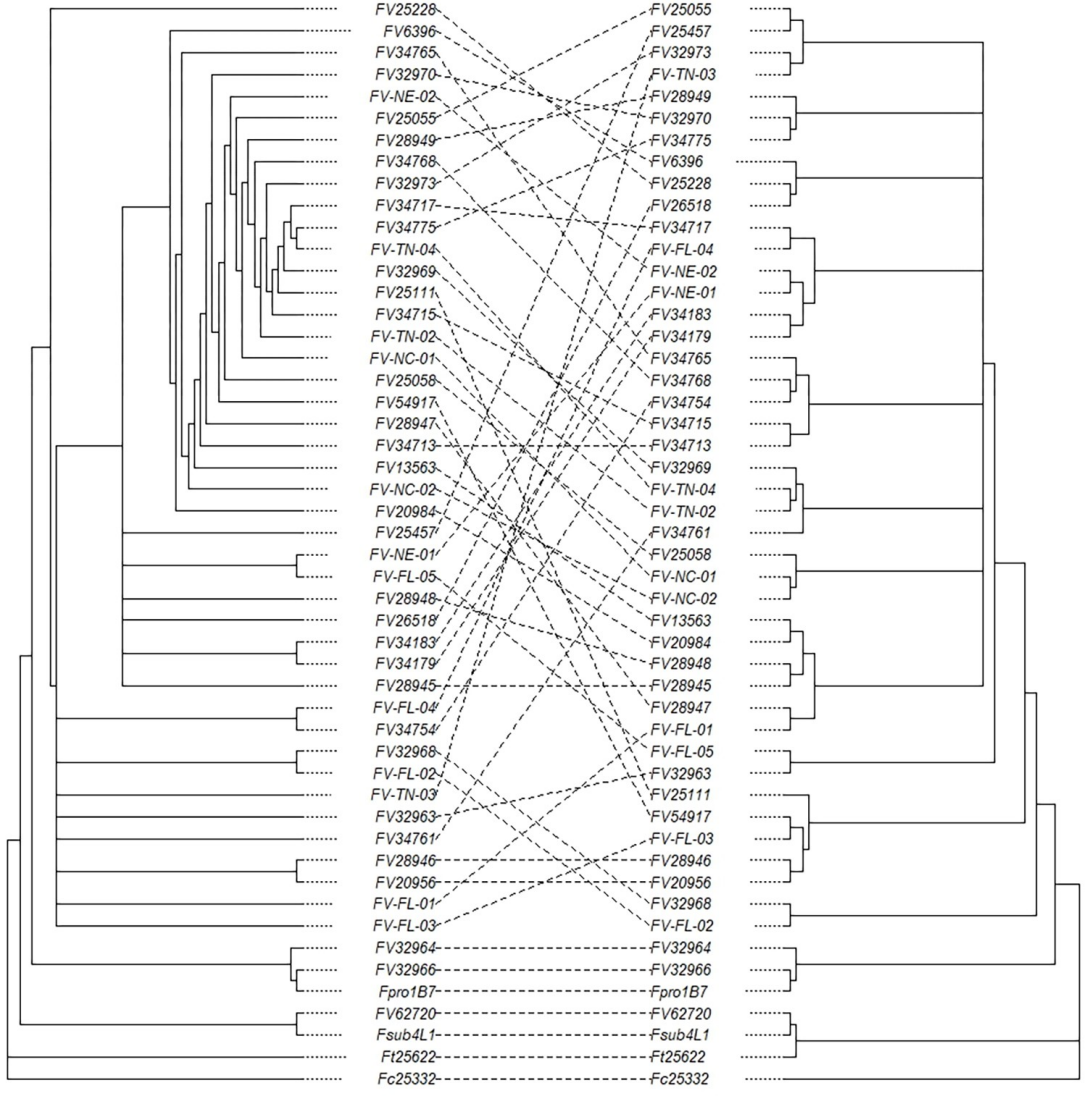
Tanglegram of putative effector and BUSCO derived phylogenetic trees. Effector derived tree is on the left and BUSCO derived tree is on the right.

We performed whole genome sequencing on 50 *Fusarium* isolates that included: 41 *Fv* isolates, 4 isolates identified as (*F*. *circinatum-*Fc25332, *F*. *temperatum*-Ft25622, *F*. *proliferatum*-Fpro1B7, *F*. *subglutinans*-Fsub4L1), and 5 unexpected out-species (FV32968, FV-FL-02, FV32966, FV32964, FV62720) that were identified following sequencing. Based on the Benchmarking Universal Single-Copy Orthologs (BUSCO) gene tree (Figs 1 and S1), isolate FV62720 is most likely *F*. *subglutinans* and isolates FV32966 and FV32964 are *F*. *proliferatum*. Of the unexpected out-species isolates, FV32968 and FV-FL-02 were placed closest to the *Fv* clade while identified as being distinct. While these isolates were closest to the *Fv* clade, several genes aligned closest to *F*. *fujikuroi* accessions from NCBI. Of the available reference genomes, Fv7600 (ASM14955v1) was the only one that has been fully annotated, with the quality of alignment scores in S1 Table reflecting this in columns presenting “contig number” and N50 statistics. The genome lengths were as expected, with the *Fv* genome ranging from 41-43Mb and the out-species being slightly larger (43-45Mb). The genome coverage ranged from 20.8x to 49.2x with the number of predicted genes (13119 to 14075) being notably higher in the out-species. Chromosome and contig names with their RefSeq ID numbers are shown in S2 Table.

### Similarities among isolates

All *Fv* isolates clustered within a single clade when either the BUSCO or the Effector genes were used to produce phylogenetic trees (Figs 1, S1 and S2). The phylogenetic tree derived from effectors had a significantly lower resolution among *Fv* isolates when compared to the BUSCO tree, which was due to the high degree of homology and conservation between effector profiles and effector sequences as well as the number of genes from which the trees were derived (786 vs. 4494). The effector-based and BUSCO-based phylogenetic trees are compared directly in the tanglegram presented in Fig 1. The tanglegram confirmed that the relationships determined from putative effector profiles mirror those of the BUSCO profiles in their ability to differentiate isolates at a species level, but they do not provide sufficient resolution for clear differentiation within species-clades. There were no disagreements between what isolates belonged in which clades and no differences in the classification of out-species clades. The lack of resolution between *Fv* isolates in the effector tree is evident given the bootstrap confidence estimates. Many isolates contained identical effector profiles, so arrangement of those isolates was done in alphabetical order. The same was true for BUSCO alignments, however fewer isolates were identical, and this provided greater resolution in the classification of isolates. We additionally compared the average nucleotide identity (ANI) across all isolates in an all-by-all manner. This provided us with similarity percentages across the isolates; for example, the *F*. *circinatum* isolate was, on average, 88.4% similar to the *Fv* clade (S3 Table). Two heatmaps were drawn from the ANI data, the first included all the isolates while the second included the 43 isolates included in the *Fv* clade so that a better differentiation could be made between highly similar isolates (S3 and S4 Figs).

### Relationships between toxin content and disease severity

**Fig 2:**
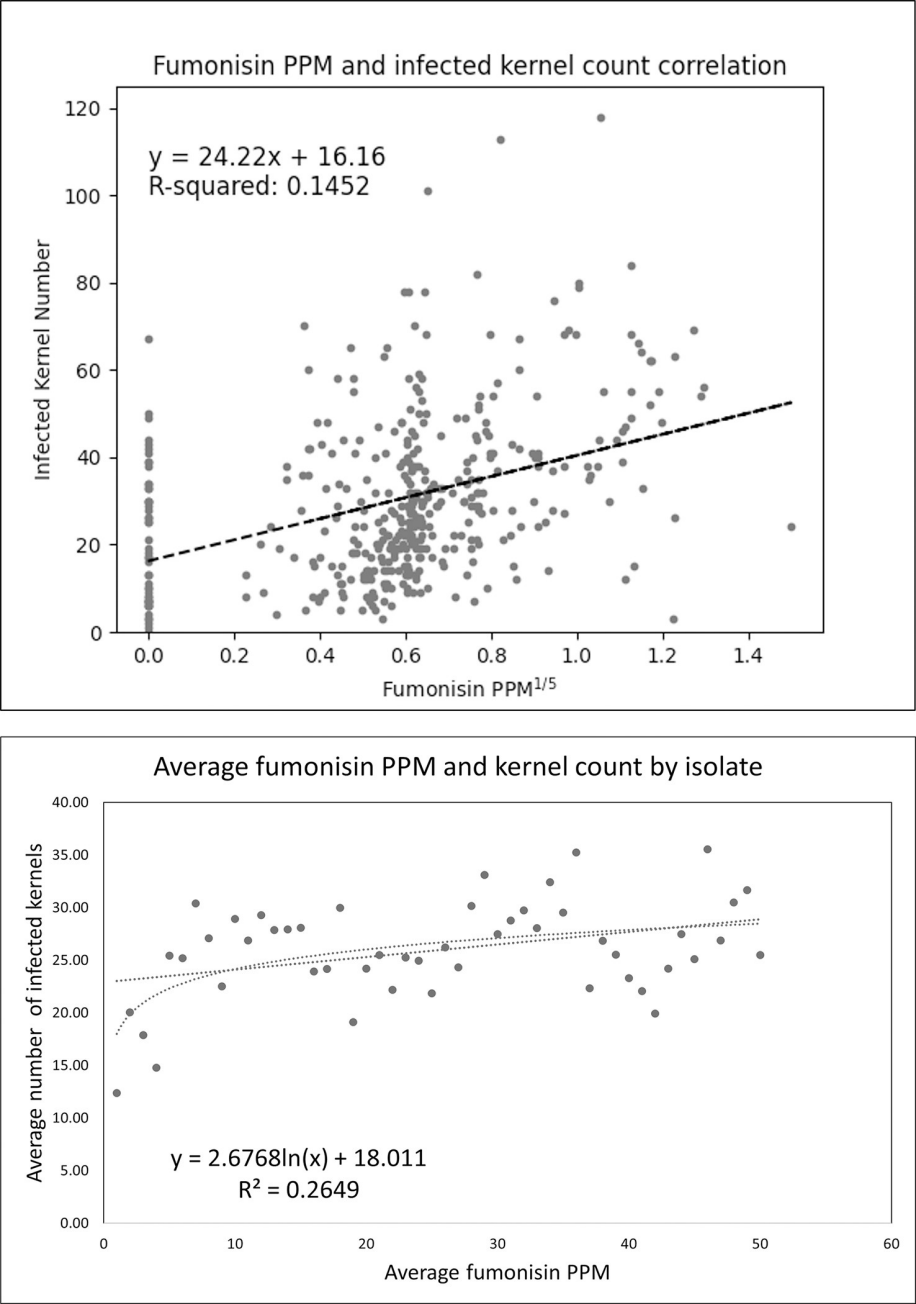
Correlations between fumonisin PPM and infected kernel counts. A. Scatterplot displaying estimates for all ears measured for fumonisin concentration, with fumonisin PPM^1/5^ presented on the X-axis and the same ear’s infected kernel count number is on the Y-axis. B. Scatterplot displaying average fumonisin PPM and average infected kernel number for each *Fusarium* isolate. The line of best fit equations and R-squared values are displayed on each plot, with Fig 2B providing two regressions and equations for the linear (right) and logarithmic (left) relationships.

Fumonisin concentrations were plotted and used to estimate correlations with disease severity. Disease severity scores (infected kernel counts) are displayed in S5A and S5B Fig, and though slightly skewed to the left, the distributions were nearly normal (Shapiro-Wilk normality test p-value = 0.3625). Correlations were first estimated between each individual ear’s fumonisin PPM and that ear’s infected kernel number (Fig 2A). The positive correlation of r = 0.38 indicated that 14.52% of the variation in fumonisin concentration was explained by the number of infected kernels. There were two clusters of fumonisin concentrations in the scatterplot, with the first identifying infections that had zero fumonisin contamination (X = 0) and a range in the number of kernels infected. The second cluster identifies ears with fumonisin concentrations around 0.6 ppm^1/5^ (0.07 ppm), which appeared to be a common contamination level for the inoculation and incubation time. This correlation was similar to the Spearman’s correlation coefficient of 0.41 for the same data, done to adjust for non-normality. The scatterplot in Fig 2B used the average fumonisin PPM and the average infected kernel number by isolate. Two correlations were calculated for this dataset, where the first was a linear correlation (r = 0.415) and the second was from a logarithmic regression (r = 0.515). These were again similar to the Spearman’s correlation coefficient of 0.46.

In addition to fumonisin concentration measurements, sequences of the 16 genes involved in the *FUM* biosynthetic gene cluster were identified in the isolates using BLAST to determine the presence, absence, or similarity of each gene for all isolates. In S6A Fig, the heatmap shows the similarity of each gene, where the darker brown colors indicate a greater similarity between the isolate’s gene sequence and the gene from the *Fv* reference. Isolates Fpro1B7, FV32964, and FV32966 (thought to be *F*. *proliferatum)*, showed, on average, an 81% similarity in genes that were present and the absence of 3 genes (*FUM8B*, *FUM11*, and *FUM18*). Four isolates lacked all genes in the *FUM* cluster, which was expected because no previous studies had detected fumonisin production in those species (*F*. *subglutinans* (FV62720 included), *F*. *circinatum*, and *F*. *temperatum*). Two isolates (FV32968 and FV-FL-02) contained only two *FUM* genes (at 96% identity) and lacked the other 14 genes. In S6B Fig, a scatterplot displays the correlation between the disease severity and the average similarity percentage of the *FUM* cluster. While the correlation was r = 0.621, isolates containing all the genes in *FUM* showed a large range in the disease severity estimates derived from kernel counts.

### Comparisons of genome size and composition

**Fig 3:**
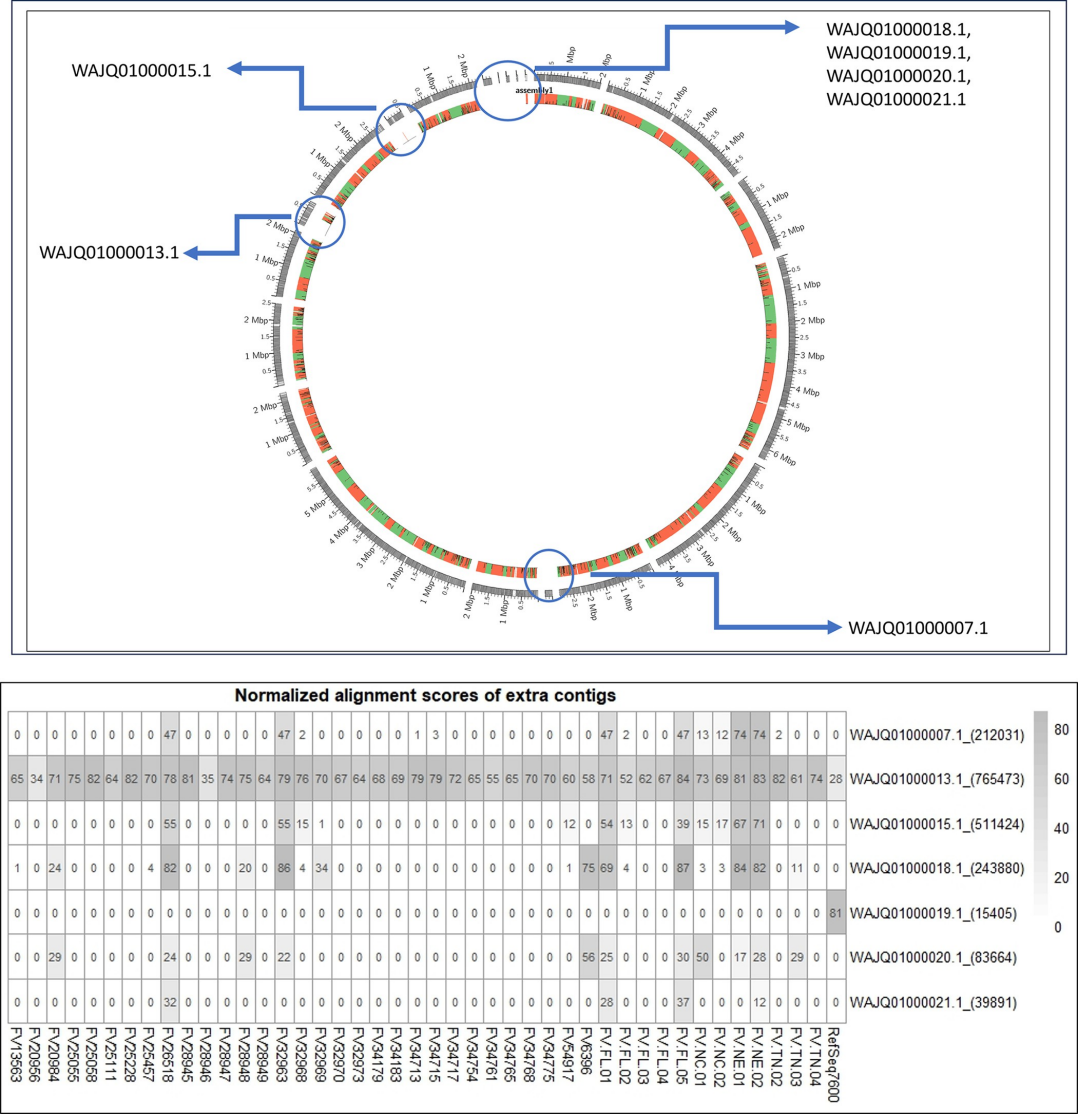
Investigation of large (>10kb) non-chromosomal contigs. A. Circos plot between reference genomes Fv7600 and Fv10027_ITA. Reference Fv10027_ITA is on the outside, Fv7600 is the interior ring. Blue circles name and indicate contigs present in Fv10027 but are absent in Fv7600. Orange blocks refer to differences in alignment and green blocks are identical alignments. **B.** Heatmap displaying all *Fv* isolates’ normalized alignment scores of the extra contigs in the Fv10027_ITA reference. Scores range from 0–100 and are displayed in each respective cell. Reference Fv7600 is also included (“RefSeq7600”).

As previous studies have shown significant genome size differences among *Fv* isolates and have proposed the presence of a twelfth chromosome [49,50], further investigation was undertaken using two different *Fv* reference genomes differing in that regard. All the *Fv* isolates were scaffolded using the two reference genomes independently, genes were predicted, and RagTag alignment statistics were summarized (S7 Table). The first reference genome (RefSeq sample Fv7600) has a genome size of 41.8 Mb, 11 chromosomes, and 36 scaffolds. The second reference genome assembly (Fv10027_ITA) was 44.6 Mb consisting of 20 contigs. In Fig 3A, a Circos plot is shown with Fv10027 as the outermost ring with Fv7600 aligned to it. Several regions are highlighted to show the absence of these contigs in Fv7600. When the order is reversed (S7 Fig) no such gaps are seen in the alignment, suggesting that these regions are present in Fv10027 but not in Fv7600. BUSCO genes were again predicted for all isolates after scaffolding with both reference genomes independently to see if a significant difference in the absent regions existed. The average number of BUSCO genes increased by one (range 0–2) when aligned to Fv10027 compared to Fv7600 and decreased by one (range 0–3) when BUSCO genes were predicted without scaffolding (S1 Table). Comparing scaffolding statistics produced by RagTag, isolates scaffolded using reference Fv10027 placed an additional 100–1400 Kb when compared to Fv7600 while scaffolding using Fv7600 reduced the number of gap base pairs (bps) from 1200–23000 when compared to Fv10027. Because the increase in placed bps was significant when using Fv10027 as the reference, we predicted genes and repeating elements from the extra regions to determine if they harbored novel functions. Fig 3B provides a heatmap of all isolates’ normalized alignment scores for each of the regions that were absent in Fv7600 with contig names and lengths included using the Fv10027 assembly. Contig WAJQ01000013.1 was the only extra contig present in all isolates, with normalized alignment scores ranging from 24% to 85%. Augustus predicted 299 genes from the Fv10027 regions missing in Fv7600, but when these regions were BLASTed against the Fv7600 Augustus gene prediction, every single gene was accounted for at a 93% or higher identity. This confirmed that while the alignment of Fv7600 had a significant number of missing regions, the genes were either copies of genes in other chromosomes or they were simply transposed. Transposable element (TE) prediction of the extra contigs was completed with the expectation that a greater number of TEs would be present if those regions were lineage specific or supernumerary chromosomes. The regions were shown to be nearly identical, if not a slightly lower TE percentage in the core chromosomes. TE percentages ranged from 0.9% to 1.29% in the extra contigs and ranged from 0.95% to 1.44% in the core genome, changing proportionally. Because no functional differences could be identified, any software requiring a reference genome and any reference to chromosome names were subsequently completed using Fv7600 as the reference to maintain consistency with the literature.

### Content and distribution of effectors, variants and CAZymes

Effectors were predicted for all isolates to determine their coordinates and to identify relationships between the total number of effectors and virulence estimates. The putative effectors of all isolates within the *Fv* clade were plotted in S8A Fig and show the distribution and density of effectors across the genome. High effector density was evident on chromosomes 2, 8, and 10 (NC_031676.1, NC_031682.1, NC_031684.1) and disproportionately high effector totals were found on chromosomes 8 and 10. Though both chromosomes are smaller than 3Mb (2.8 and 2.2Mb respectively), they contained the greatest number of effectors (2235 and 2440 respectively). S8B Fig shows a heatmap arranged from the greatest to the smallest number of effectors per isolate. *F*. *subglutinans* (Fsub4L1) had the greatest number of effectors (543) and *F*. *temperatum* (Ft25622) had the fewest (492), while the total number of unique effectors identified across all isolates was 786. To incorporate disease severity estimates into our comparative genomic framework, the average number of infected kernels per isolate were correlated with the number of effectors in that isolate. The correlation coefficient was nearly zero (r = 0.001), supporting the idea that specific effectors rather than total effector number regulate the severity of FER disease symptoms.

**Fig 4:**
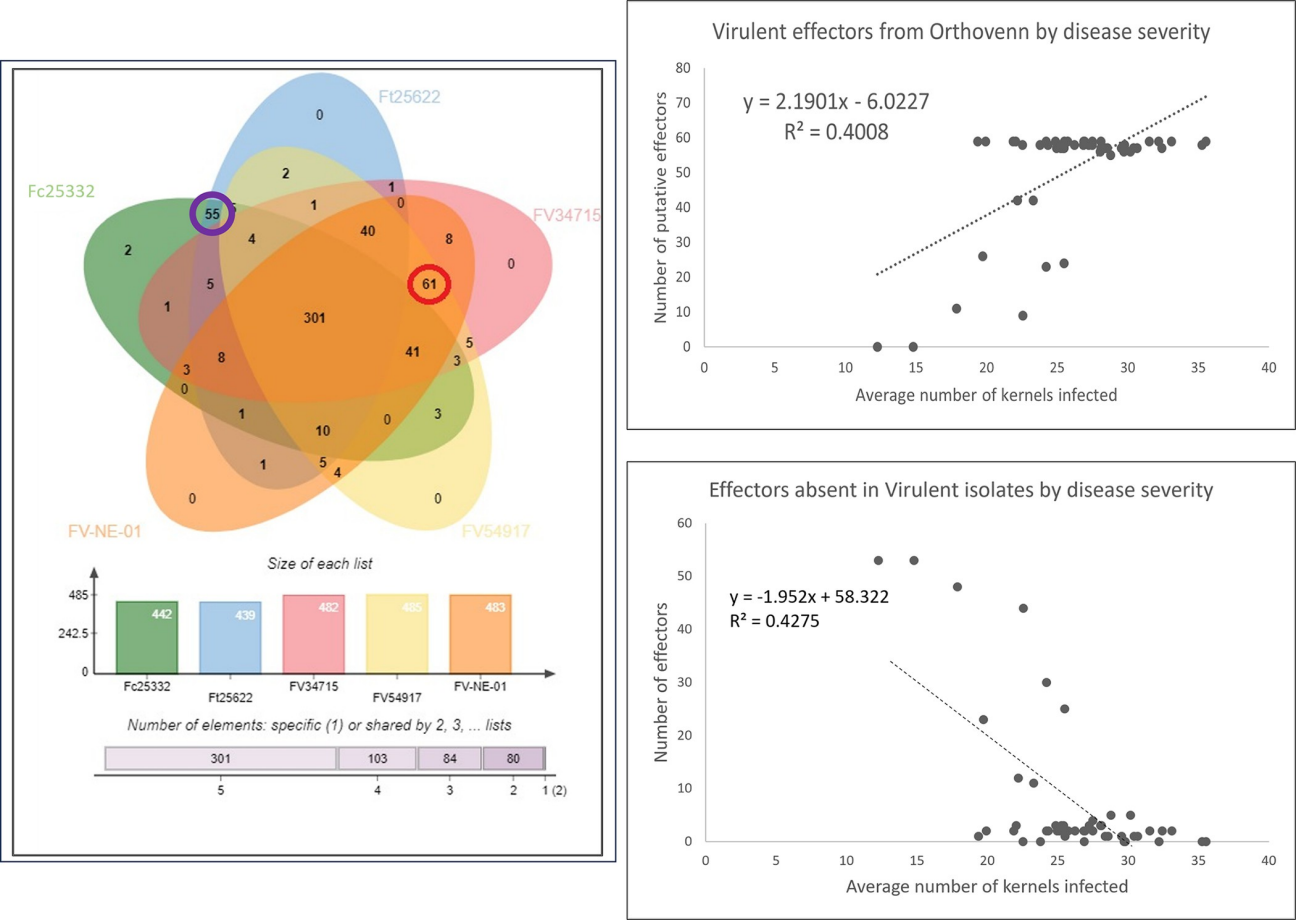
Specific effector orthogroup selection. A. OrthoVenn3 display of effector orthogroups between five isolates. Effectors from two isolates with low disease severity and three isolates with high disease severity were compared to determine unique effectors. The red circle indicates the number of unique effectors between the highly severe isolates and the purple circle indicates the number of effectors unique to the least severe isolates. B. Scatterplot between effectors from severe isolates and disease severity. The effectors unique to the three most severe isolates are on the Y-axis, and the average number of kernels infected by each isolate in on the X-axis. C. Scatterplot between effectors from the least severe isolates and disease severity. The effectors unique to the two least severe isolates are on the Y-axis, and the average number of kernels infected by each isolate in on the X-axis.

As the total number of effectors per isolate was unrelated to disease severity, we sought methods to narrow down the pool of effectors to identify those that may contribute to disease severity. We used OrthoFinder (illustrated in Fig 4A using OrthoVenn3) to cluster the effectors of the three most severe (FV34715, FV54917, FV-NE-01) and two least severe (Fc25332, Ft25622) isolates. This yielded two new sets of effectors with 55 unique to the least severe isolates and 61 unique to the most severe isolates (Fig 4A). Each new effector set was then correlated with disease severity scores and plotted (Fig 4B & 4C) with significant correlations (r = 0.633) for the effectors present in more severe isolates and for the effectors that were absent in more severe isolates but present in the less severe isolates (r = 0.654) (Fig 4B and 4C). Using the subset of effectors that were present or absent in the most severe isolates, there was significant clustering in the scatterplots with isolates in the *Fv* clade grouping together regardless of disease severity estimates. Given the lack of resolution within *Fv* isolates, we selected two isolates for more detailed comparisons.

**Fig 5:**
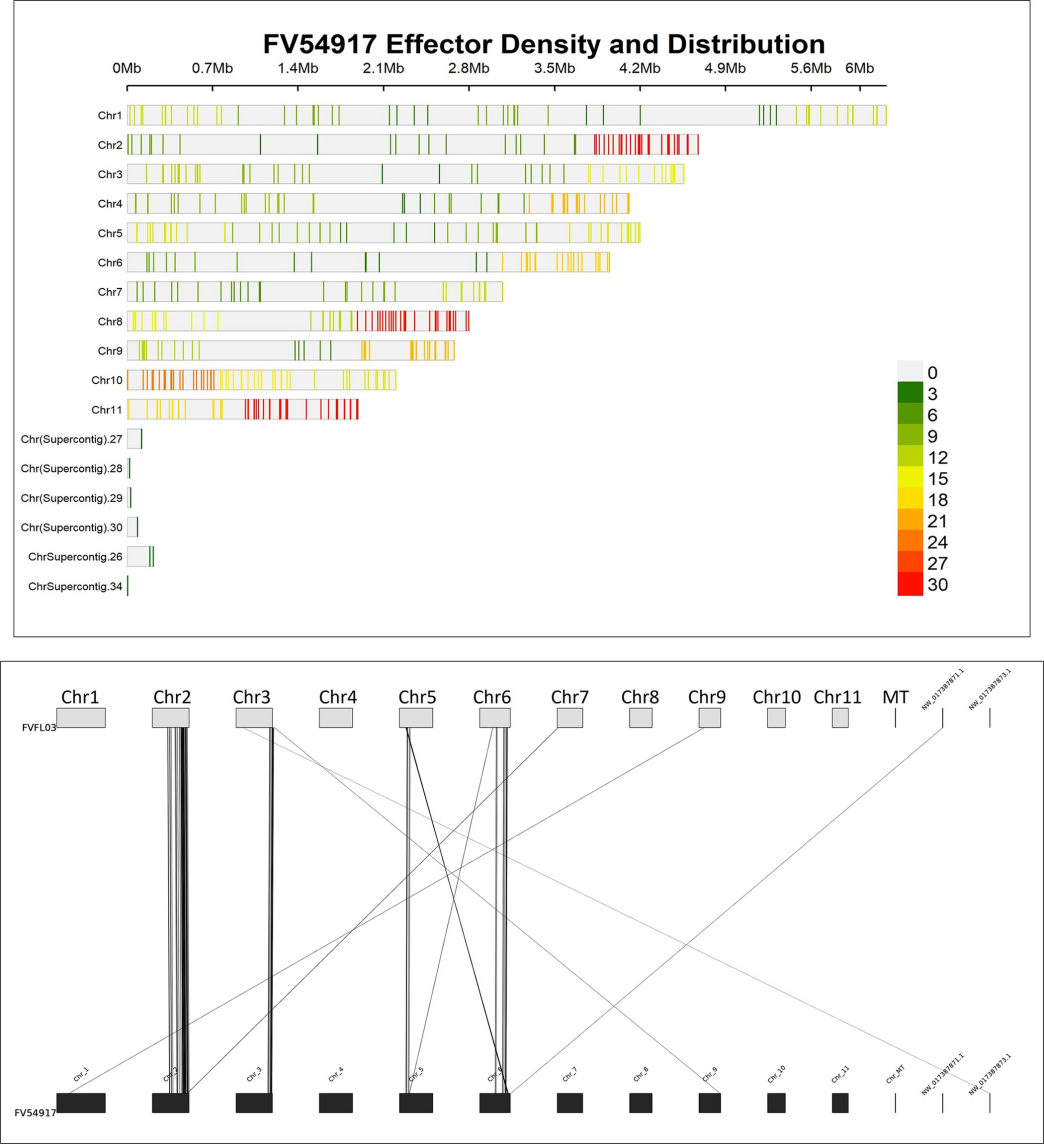
Effector distribution and movement between FV54917 and FV-FL-03. A. Density and distribution plot of effectors from high disease severity isolate FV54917. B. Synteny plot of effectors which had a positional difference greater than 100kb between FV-FL-03 and FV54917. Lines represent individual effectors and their positional changes between the two isolates.

The two most genetically similar *Fv* isolates with contrasting disease severity estimates were FV54917 and FV-FL-03. Phylogenetic trees (Fig 1) indicated that these isolates were closely related, with FV54917 providing the second highest disease severity estimate and FV-FL-03 providing one of the lowest (S5A Fig). Of FV54917’s 527 effectors and FV-FL-03’s 533 effectors, only 36 were either present or absent when the opposite was true in the other isolate. The density and distribution of FV54917’s effectors displayed in Fig 5A are slightly different than total isolate effector distribution discussed above (S8A Fig). The effector amino acid sequences searched for in the UniProt protein database identified 29 of the 36 effectors that had either conserved domains or putative functions annotated (Table 1). Of the 491 effectors that the two isolates shared, 79 showed a change in position greater than 100kb, and 12 were located on different chromosomes. The sequences of these 79 effectors were BLASTed to determine if there was any known function, and those annotations are displayed in S5 Table.

**Table 1:** Effectors of isolates FV54917 and FV-FL-03 when one is differentially present or absent compared to each other. Under each isolate, 0 indicates absence while 1 indicates presence of the effector. Gene type or conserved domain of the predicted effector from BLAST results and the reference to that gene are also listed.

Effector group	FV54917	FV-FL-03	Gene type or conserved domain	Gene reference
OG0000002	0	1	LysM domain containing protein	W7M937
OG0000342	1	0	IgE-binding protein	W7M5Y0
OG0000368	0	1	Phosphoglycerate mutase	W7MX34
OG0000407	0	1	GH64 domain-containing protein	W7MQY6
OG0000428	1	0	Extracellular membrane protein CFEM domain-containing protein	W7MIG6
OG0000447	1	0	Lysine-specific metallo-endopeptidase domain-containing protein	A0A139YBB4
OG0000455	0	1	Endo-1,4-beta-xylanase	W7N504
OG0000470	1	0	Extracellular membrane protein CFEM domain-containing protein	W7LBX2
OG0000484	1	0	WSC domain-containing protein	W7M8M1
OG0000495	0	1	Ig-like domain-containing protein	W7LQG4
OG0000503	0	1	Peptidase A1 domain-containing protein	W7MLQ6
OG0000504	1	0	Jacalin-type lectin domain-containing protein	W7MMN7
OG0000505	0	1	Ecp2 effector protein domain-containing protein	W7N7V3
OG0000508	0	1	Cyanovirin-N domain-containing protein	W7MSM9
OG0000510	0	1	alpha-galactosidase	W7NHJ2
OG0000511	0	1	Chitin-binding type-4 domain-containing protein	W7MAL2
OG0000512	1	0	Ecp2 effector protein domain-containing protein	W7MJZ2
OG0000522	0	1	Extracellular membrane protein CFEM domain-containing protein	W7MW51
OG0000531	0	1	Heme haloperoxidase family profile domain-containing protein	W7MRU9
OG0000536	1	0	Cutinase	W7MRH0
OG0000545	0	1	Pectate lyase domain-containing protein	W7MN59
OG0000547	0	1	Ig-like domain-containing protein	W7MSZ4
OG0000555	0	1	Ig-like domain-containing protein	W7MUX9
OG0000563	0	1	CBM-cenC domain-containing protein	W7MVY8
OG0000564	0	1	Glucanase	W7MN55
OG0000611	0	1	Arabinosidase	W7N780
OG0000637	0	1	Glucanase	W7MWM3
OG0000672	0	1	Apple domain-containing protein	W7LSJ4
OG0000718	0	1	Fucose-specific lectin	W7MDA4

Variants that were differentially present or absent between the two isolates and were predicted to have a “high impact” on gene function were pulled out for each isolate, resulting in 62,958 for FV54917 and 58,439 for FV-FL-03. The genes and the proposed impact of the variant are presented in S6 Table along with the chromosome and position where the variant occurred with a total of 40 protein variants identified in the isolates. The location and density of the unique variants for each isolate are shown in S9A and S9B Fig.

Carbohydrate-active enzymes (CAZymes) were also identified and correlated with disease severity estimations. S10A–S10F Fig show the correlation of disease severity with each CAZyme class, the highest correlation being with the classes “Auxiliary Activities” (r = 0.420) and Carbohydrate esterase (r = 0.393).

### Isolate origin and virulence

Finally, the substrate and geographic origins of each isolate were used to investigate the differences in the disease severity scores. Eight isolates lacked information about the original host and seven had no information regarding geographic origin. No patterns were seen between the geographic origin and the virulence of the isolates. Substrate data of each isolate was divided into two groups: maize and not maize. S11B Fig displays a boxplot comparing disease severity between the two original substrate groups and two tests of significance. The T-Test significance value = 0.050 suggested that isolates coming from maize produced significantly larger lesions and or infected more kernels.

## Discussion

### Disease severity and toxin production

The species of *Fusarium* that cause Fusarium Ear Rot (FER) in maize are highly variable in their ability to infect, cause damage, produce toxins, and reduce maize yield. While the primary causal agent of FER is *Fusarium verticillioides* (*Fv*), several other *Fusarium* species are found in infected maize ears. The availability of a diverse set of *Fusarium* isolates with sequenced genomes provided a means to conduct both intraspecific and interspecific comparative genomic studies for a pathosystem that has been associated with quantitative disease resistance. Because of the extensive genomic homology between *Fusarium* species, the quantitative nature of the FER pathosystem, the large number of pathogenicity-associated genes, and the availability of disease severity data from controlled inoculations, we present this comparative genomic analysis of 50 *Fusarium* isolates as a means to identify genetic mechanisms controlling disease severity.

Initially, only four isolates were considered alternative species and were included to ensure differences in genome composition and disease symptom expression for comparisons with *Fv* isolates. Our preliminary genome assembly and phylogenomic comparisons determined that several isolates differed from their listed species names. Isolate FV62720 is likely to be *F*. *subglutinans*, while FV32964 and FV32966 are likely *F*. *proliferatum* isolates. Two isolates, FV32968 and FV-FL-02, were placed outside of the confirmed *Fv* clade and are potentially members of the *Fusarium fujikuroi* species complex. The pine (*Pinus* sp.) pathogen *F*. *circinatum* (Fc25332) provided a reliable and distinct phylogenetic root as the most distantly related of the out-species that has been shown to infect maize and produce little to no disease severity [41]. Using infected kernel counts, Fc25332 consistently showed the lowest disease severity of all the isolates, confirming previous findings. Although the putative effectors did not provide sufficient information to differentiate *Fv* isolates to the same degree as the BUSCO-based tree, the effector tree (S2 Fig) did mirror the clades and species-level differentiation was possible with Fc25332 shown to be the most distantly related isolate in both comparisons. This can be seen in the tanglegram (Fig 1), with the alternative species clearly differentiated and the order of the *Fv* isolates lacking alignment across trees due to insufficient information provided by the sequences from the putative effectors. The identical species level designations show the potential of using the effectorome (all putative effector genes) for the simultaneous identification of species using pathogenicity-related genes that may also allow for the differentiation of virulent and avirulent isolates. The same clade-level differentiation can be seen in the average nucleotide identity (ANI) scores across the isolates and the trees on the ANI heatmaps (S3 and S4 Figs). The ANI scores additionally confirmed the species level differentiation seen in the BUSCO tree and provided a numerical comparison between individual isolates (S3 Table). Isolates FV32968 and FV-FL-02 were 97.5% similar to all true *Fv* isolates, compared to the average 99.07% of the *Fv* isolates compared to that clade. Out-species ranged from 88.3% (*F*. *circinatum*)– 93.8% (*F*. *temperatum*) identity compared to all other isolates.

Phenotyping inoculated ears by counting visually infected kernels provided large quantitative differences in disease severity and differentiation among isolates. Due to the variability in phenotyping methods available for the FER pathosystem and the practical importance of fumonisin contamination in the food supply, we validated relationships between our kernel-based phenotyping method and measurements of fumonisin levels. The correlations between kernel counts and fumonisin measurements for all phenotyped ears yielded a low but positive correlation (r = 0.381). The correlation was slightly higher when averages of infected kernel counts and of the fumonisin measurements were taken (r = 0.415), while a logarithmic regression increased the correlation between disease symptoms and fumonisin content to r = 0.515. While the correlations in the literature range greatly, on average, our correlations are slightly lower than those found in the literature [51–53]. We believe the correlation is insufficient to justify using visual severity scores as a proxy for fumonisin measurements and subsequent breeding efforts if any significant genotypic diversity is present in either population. The impact of the genotypic diversity of both *Fusarium* isolates and maize varieties should be considered when making these comparisons. Our use of 170 unique maize genotypes specifically chosen to maximize genotypic diversity along with 50 *Fusarium* isolates, six of which should not have been able to produce any fumonisin at all (S6A Fig), makes direct comparisons infeasible. While these isolates did produce fumonisin levels that were lower than the other 44 *Fv* isolates on average, some still produced small amounts of FUM where genes in the FUM pathway were absent. This suggests some experimental or external sources of error were present in the ELISA estimated fumonisin concentration data.

Introducing additional sources of variance in the form of diverse maize varieties and *Fusarium* isolates is expected to increase the observed phenotypic variation. For example, phenotyping ears from a single maize variety with a single *Fusarium* isolate should produce a more reproducible and less variable estimate of fumonisin content. Conversely, the inclusion of a more disease resistant variety may lead to reduced disease severity while fumonisin production is similar. Likewise, a single *Fv* isolate may produce a high concentration of fumonisin in a particular maize variety without causing high disease severity. These data, taken with the improved logarithmic correlation, suggest that using infected kernel counts is a problematic and non-linear method of quantifying fumonisin levels. Although unable to be concluded from these data, it is likely that visual estimates of disease severity should not be used to measure fumonisin content when genotypic diversity is present. Reduced scales measuring severity (i.e. 1–10) are also likely to inflate the correlation compared to using a raw scale such as kernel counting which ranged from 1–110.

As expected, all out-species were in the lower half of the virulence scores (S5A Fig), although FV32966 and FV32964 are thought to be the same species as Fpro1B7 (*F*. *proliferatum)* and they differed in disease severity considerably, ranking 28^th^, 37^th^, and 46^th^, respectively. This is perhaps explained by the host range of *F*. *proliferatum*, which includes a wide range of plant species with isolates developing adaptations that vary in disease severity across different hosts. Evidence contrary to this hypothesis is that Fpro1B7 was isolated from a maize plant while FV32964 was isolated from human blood. Results from the T-test between isolates from maize or non-maize hosts indicated a slightly significant association between isolate host and disease severity (T-test p = 0.05 (S11B Fig)), providing evidence that *Fusarium* can adapt to their hosts to cause higher disease severity and *Fv* isolates cause more severe maize infections compared to other *Fusarium* species that are also found in maize.

In the literature on *Fv* and other *Fusarium* species, evidence suggests that a twelfth chromosome is present in some isolates [47,50]. As we had access to 43 *Fv* genomes (FV-FL-02 and FV32968 were included), we investigated this theory by comparing two different reference genomes, Fv7600 and Fv10027_ITA. Fv10027 was larger by 1.6 Mb and contained 7 extra contigs, 3 of which were over 200 Kb compared to Fv7600. After identifying these extra contigs by scaffolding each reference with the other reference and providing comparisons as Circos plots (Figs 3A and S7), there was sufficient evidence of extra-chromosomal regions in Fv10027 and several of our isolates (Fig 3B). These extra-chromosomal regions have many names (lineage specific, conditionally dispensable, supernumerary, accessory, mini, or B-chromosomes) and are best studied in the forma speciales of *F*. *oxysporum*.

In 2010, Ma et. al. investigated these regions, referring to them as lineage-specific (LS) genomic regions [47]. They found that in *F*. *oxysporum* f. sp. *lycopersici*, LS regions occupied four full chromosomes, had a high density of transposons, had genes related to pathogenicity, and could be transferred horizontally to convert non-pathogenic strains into pathogenic strains. Following this methodology, we identified genes and repeat sequences in the extra chromosomal regions and compared them to the core chromosomes to determine if there was evidence to consider these extra contigs as LS genomic regions. Not only did the extra contigs contain a lower average percentage of repetitive sequences than the core chromosomes (1.11% vs 1.23%, respectively), but all the 299 predicted genes in the extra contigs were identified in the Fv7600 reference at an identity of 93% or higher. Also, there was no correlation between the presence of extra contigs and disease severity for each isolate (r = 0.06). Given these data, we do not consider these regions to be similar in function to the LS regions identified in *F*. *oxysporum* f. sp. *lycopersici*. Although they are significant in size, they do not contain any functional genes that are absent in the core chromosomes of Fv7600. Because of these factors and the RefSeq status of Fv7600, we chose to scaffold and use Fv7600 as the reference whenever necessary.

### Comparisons of *Fv* genome composition

One group of phytopathogen genes known to regulate disease severity are effectors. Effectors can act as virulence or avirulence factors depending on the current state of the molecular arms race between host and pathogen. The “zig zag model” describes this phenomenon well, where pathogen effectors that are unrecognized by the host can contribute to disease, but effectors recognized by the host (then termed avirulence genes) can trigger the defense response characteristic of effector triggered immunity (ETI) [54]. Hemibiotrophs, such as *Fusarium* spp., evade or repress the plant immune system before deploying effectors when they switch to a pathogenic lifestyle [55,56]. Because effectors can act as pathogenicity factors, avirulence factors, or neither, we investigated the correlation between groups of effectors and disease severity. Putative effectors’ density and distribution across the *Fv* genome (Fig 5A for FV54917 and S8A Fig) showed chromosomes 8 and 10 contained the most predicted effectors while being two of the shortest. No correlation was identified between effector number and disease severity (r = .001). This was the expected result, showing that effectors do not act additively to cause disease severity. Identifying effectors that may actively contribute to disease severity by clustering effector groups from isolates with contrasting virulence levels revealed that 301 of the predicted effectors were shared across the chosen isolates, 61 to be present in only the most severe isolates, and 55 in the least severe isolates. This search for effectors provided further evidence that FER is highly quantitative with many alleles contributing variable degrees to disease severity. Of the effectors that were similar to a reference protein, several were of particular interest due to their known roles in *Fusarium* pathogenicity: pectate lyase and acetylexylan esterase in the high-severity effector group; cutinase and glucuronoxylanase in the low-severity effector group [57–60].

Though these methods led to a more specific list of potentially significant effectors, using out-species isolates rather than low-severity *Fv* isolates had drawbacks. First, the effectors unique to the out-species isolates do not necessarily have direct implications about the effectorome of *Fv*. Fortunately, using two different out-species limits the number of non-maize-specific effectors. For example, effectors unique to *F*. *circinatum* that may contribute to disease on *Pinus* spp. should not be present in *F*. *temperatum* (the other out-species and does not infect *Pinus*) and the identified effectors are likely conserved across additional *Fusarium* spp. A second drawback is that because no *Fv* isolates representing low disease severity were included, narrowing the list of their effectors was limited. This was done because when multiple low-severity *Fv* isolates were used there were either no unique effectors, or they were inconsistent. To address this, we narrowed the comparison to two closely related isolates with contrasting levels of virulence.

We chose the contrasting isolates FV54917 and FV-FL-03, ranking 2^nd^ and 43^rd^ in disease severity, respectively, to identify effector and variant differences that may be associated with FER severity. We chose these isolates based on their close phylogenomic relationship (as seen in the BUSCO-based tree in S1 Fig) and distinct disease severity scores. Table 1 displays the effectors that are unique to each isolate with a reference gene or conserved domain. FV54917 had 8 unique effectors with a database reference and FV-FL-03 had 21. Several of the proteins encoded from the effectors have previously been identified as pathogenicity related proteins such as cutinase, chitin-binding proteins, glucanase, xylanase, and other conserved domains [59,61]. Notable too were the effectors identified using both this approach and OrthoVenn3/OrthoFinder. A cell wall integrity and stress response component (WSC) domain-containing protein was present the high-severity group (Table 1) and absent in FV-FL-03 (S1 Table), while a pectate lyase A protein was present in the low-severity group and absent from FV54917. Limited literature exists on WSC domains in phytopathology; however, it has been shown to act as a substrate anchor for the enzyme in *Pyricularia oryzae* [62]. Pectate lyases, conversely, are well known pathogenicity factors for multiple fungal pathogens including *Fusarium* [57,63]. We also compared the density and distribution of these two isolate’s variants (SNPs or indels) called against the *Fv* reference genome (S9A and S9B Fig) to determine if there were significant differences. The most obvious difference was the number of variants in chromosome 5, with FV54917 having 9990 variants and FV-FL-03 having only 3928. We selected a subset of the variants if they were unique between the two isolates and their effects on protein composition were rated as “high” by SNPeff. S6 Table contains a record of the position, variant effect(s), protein type targeted by the variant, and that protein’s reference for all high effect variants for both isolates. Of note were multiple splice sites identified in a pectate lyase copy in FV54917. This mutation of pectate lyase, taken in conjunction with the identification of pectate lyase in low-severity isolates and its absence in FV54917 provides supports its role as an avirulence gene.

Various pectate lyase genes were again identified when determining the 79 effectors that were present in both FV54917 and FV-FL-03 (with at least 95% identity) but with a positional difference of more than 100 Kb (Fig 5B and S5 Table). Twelve of the effectors, including a pectate lyase, were positioned in entirely different chromosomes in the two isolates. Control over transcription is more likely to vary when genes are in such different positions or have additional copies in other locations. This strategy is well known in *Fusarium* pathogenomics, where the high concentration of transposable elements allows for rapid gene diversification without the need for sexual recombination [64,65]. Theoretically, this is particularly impactful in quantitative diseases such as FER, where accumulating multiple minor changes in the expression and the sequences of non-host-specific pathogenicity genes make the pathogen more resilient and could provide a competitive advantage over other microbes.

## Conclusions

While potentially beneficial for the pathogen, the quantitative nature of disease resistance in FER also provides an opportunity to decrease mycotoxin contamination in one of the world’s most important crops, maize. Introgression of many minor alleles contributing to disease resistance into maize varieties is more difficult and time consuming than incorporating a single major gene (qualitative resistance) but is expected to provide a more durable form of resistance. In this research, we attempted to use comparative genomics and a quantitative phenotype (disease severity) to identify fungal alleles that may be involved in FER pathogenicity. Disease severity showed a low correlation with the levels of fumonisin contamination, likely due to the large genotypic diversity of the host and pathogen populations used in this study. While researchers still use visual disease severity as a proxy for fumonisin levels due to the high cost and low efficiency of chemical testing fumonisin levels, we suggest that these chemical methods should be used to reduce error in analysis. Alternatively, other technologies such as machine vision algorithms in conjunction with multi-wavelength spectroscopy may be used to produce higher correlations with fumonisin levels, and those tools should also be evaluated on diverse populations before being deployed.

Several gene groups were analyzed in this research by comparing low disease severity isolates with high disease severity isolates to identify putative genes and proteins that were differentially present or absent. Frequently, when one or several alleles were identified in a virulent isolate, they were found to have a varied presence across multiple other virulent isolates, again confirming the quantitative nature of FER and confirming that transcriptomic, gene knock-out, or other experimental approaches will be required to validate gene targets. We repeatedly identified gene copies of pectate lyase as a potential avirulence gene that could code for proteins recognizable to the host and capable of inducing a defense response.

The identification of specific loci involved in pathogenicity may be improved by using further bioinformatic and statistical approaches and combining these genomic data with other phenotyping methods and transcriptomics. The inclusion of these genomic data along with the disease phenotypes in the public domain along with the identification of virulence mechanisms is intended to support maize breeding programs to produce varieties that will reduce fumonisin contamination of maize and their associated impacts on health.

## Materials and methods

### Obtaining and sequencing Fusarium isolates

The 50 isolates consisted of 46 isolates believed to be *Fv*, and one isolate each of *F*. *circinatum*, *F*. *temperatum*, *F*. *proliferatum*, and *F*. *subglutinans*. Each of these “out-species” has been isolated from infected maize in previous studies. The isolates were primarily sourced from the USDA-ARS Culture Collection (NRRL) and others were donated from across the United States by external researchers or were isolated from maize plants locally. Isolate names were retained for all NRRL isolates. Isolates were cultured on potato dextrose agar (PDA) or isolated directly from plant material when collected from the field. To ensure a single genotype was represented, a single spore was extracted from each isolate and grown on a PDA plate before storing at -80°C. Before whole genome sequencing, a PCR assay was performed on all isolates that were not sourced from the NRRL using primers FV-F2/FV-R from Faria et al (2011) [66] to confirm their identity as *Fv*. Isolates were then grown on PDA plates for 14-days before the mycelial mat was scraped off, placed in a 2ml tube, and lyophilized. Lyophilized tissue was then placed into a 96 well plate and sent to Rapid Genomics LLC, Gainesville FL, for extractions and Illumina pair-end whole genome sequencing. Isolates were obtained under the APHIS permit number P526P-21-07097.

### De novo assembly, alignment, annotation, and gene/protein prediction

Unpaired FASTQ files were initially trimmed and assessed for quality using Trimmomatic and FastQC respectively [67,68]. Trimmed reads were assembled de novo using Spades V3.9.0 into FASTA files [69]. Using RagTag, assemblies were scaffolded and patched using two reference genomes independently, *Fusarium verticillioides* 7600 (GCF_000149555.1) and Fv10027_ITA (GCA_020882315.1) for all *Fv* isolates [70]. Other species were scaffolded to previously assembled genomes of their respective species (S1 Table). Next, BUSCO v4.0.6 was used for BUSCO gene identification in order to assess genome completeness using the “hypocreales_odb10” dataset [71]. Based on low quality genome completeness for some isolates, phylogenetic trees created using the Basic Local Alignment Sequencing Tool (BLAST) to align BUSCO genes were used to identify suspect isolates. Five isolates initially identified as *Fv* were subsequently identified as other species and were re-assembled, re-aligned, and genes were re-identified using the reference genome that was closest to their species (S1 Table). Augustus V3.4.0 was then used for gene prediction of all genomes with “species = Fusarium” [72].

To obtain additional assembly statistics, Bowtie2 was used to align reads into contigs and chromosomes in SAM and BAM formats and samtools was used to view those statistics (S1 Table) [73]. Freebayes v6.1 was used to create Variant Call Files (VCF) using the *Fv* reference for all isolates so that variants could be compared across all isolates [74]. Variants were filtered using VCFtools, the Ensembl Variant Effect Predictor tool, and SnpEff/SnpSift, where the severity of impact estimations were estimated and then viewed on the Variant Effect Predictor (VEP) website [75–77]. Alignment differences between the references were extracted and investigated using Augustus to predict all the genes in the genomes and BLASTp to determine if any were absent or significantly different. Repeat sequence and transposable element prediction were predicted using RepeatModeler and RepeatMasker [78,79]. Normalized alignment scores were calculated using -1 for misalignments and +1 for correct alignments and then adjusted for sequence length over reference length. Genes predicted by Augustus were then filtered using SignalP v5.0b to identify those with a signal peptide [80]. EffectorP was then run through Python to predict any putative fungal effectors [81]. Protein sequences of genes previously identified in the fumonisin biosynthetic gene cluster, *FUM*, were downloaded from NCBI (FVEG_00316, 317, 319–329, 14634, and 14635 in the *Fv* reference genome) and BLASTp was used locally to determine presence, location, and sequence identity in the *Fusarium* isolates. The putative effectors predicted from EffectorP were run through OrthoFinder v.2.5.2 for clustering and comparisons across all isolates [82]. The heatmap representing effector presence across isolates was created by using the table output of OrthoFinder and converting outputs into a binary presence/absence table. To predict carbohydrate-active enzymes (CAZymes), dbCAN v.4.0.0 was used with the output of SignalP, and a custom Python script was used to extract only the CAZymes that were predicted by all three tools used by dbCAN [83]. Another Python script was used to extract the table from the OrthoFinder output and convert it to a format usable for making a heatmap in R. Quast [84] was used to evaluate the quality of genomes and to compare alignment of the two different reference genomes using Circos [85] Average nucleotide identity scores were generated using pyani [86].

### Figures and descriptive analyses

The BUSCO phylogenetic tree was built using IqTree v2.1.3 and a custom python script [87]. The phylogenetic tree of putative effectors was an output of OrthoFinder. Both trees used the least similar *Fusarium* isolate (*F*. *circinatum* or Fc25332) as the root. All heatmaps were drawn in R using the “pheatmap” package [88]. The tanglegram of the two phylogenetic trees was created in R using the “cophylo” function from the “phytools” package [89]. The density and distribution plot of effector locations was also created in R using the package “CMplot”, obtaining the coordinates from the output from EffectorP. Scatterplots and corresponding statistical analyses were created and calculated in either Microsoft Excel or Python’s Matplotlib [90]. OrthoVenn3 diagrams were created using OrthoVenn3 run locally through Docker as described in the instructions. The boxplots in S11B Fig were also created locally on Python using the “matplotlib”, “seaborn”, “pandas”, and “scipy” packages.

### Methods of inoculation and phenotyping disease severity

Spore solutions were prepared by growing isolates in 1/3^rd^ strength potato dextrose broth (PDB) for 5 days while being shaken continually. Spore counts were determined using a hemocytometer and concentrations were adjusted to 5x10^5^ spores/ml. Maize plants used to produce ears for inoculations were grown in 3 seasons: Spring 2022, Fall 2022, and Spring 2023, with sixty seeds sown from a total of 250 maize varieties in Citra, FL at the University of Florida Plant Science Research and Education Unit. Each season, varieties that produced 30 or more undamaged and uninfected ears were harvested 21–24 days after the 50% silking date of that variety, reducing the number of varieties that were assessed for disease symptoms to 160. Healthy ears were brought back to a lab where they were surface sterilized, inoculated, and incubated for one week before phenotyping disease symptoms. Inoculations were performed to simulate wounding followed by the introduction of infectious spores. This was done by piercing each ear with a sterile 18G needle, then using a new needle attached to a PrimaTech Adjustible Dose Vaccinator that was loaded with 0.75ml of conidial suspension and injected through the husk into the ear. Thirty ears were used to evaluate each maize variety, receiving inoculations from ten different *Fusarium* isolates in triplicate. Inoculations were completed using a circular design that allocated each *Fusarium* isolate to ten maize varieties. Inoculated ears were placed in labelled bags and incubated for one week in a growth chamber set to have 12/12 hours of light/dark and the temperature at 30°C. Ears were then removed from the bag, husked manually, and infected kernels were counted and recorded. Images were also taken using a custom-built imaging device for subsequent image analysis and disease phenotyping. The total number of ears that were phenotyped was 3716. Additional details and information regarding methods of inoculation and phenotyping can be found in Hudson et al. (2023) [91].

### Fumonisin concentration measurements

Fumonisin concentrations were measured from 417 maize ears that were inoculated as described above in the Spring 2023 season. Ears inoculated with isolates known to have the *FUM* biosynthetic gene cluster were prioritized for sampling, and samples representing species known to not have the *FUM* cluster were minimized in sampling. Otherwise, sampling was randomly generated using the index number pre-assigned to each ear. Ears were shelled and the seeds were ground to a fine powder using a coffee grinder before fumonisin was extracted. We used the protocol as described in the Helica Fumonisin Hydro ELISA kit by Hygiena. Briefly, the ground maize was shaken in distilled water for three minutes and allowed to precipitate before the top layer was transferred to a new tube and centrifuged for five minutes. The supernatant was transferred to a new tube and then diluted. The ELISA test was performed following the manufacturer’s instructions and the optical density (OD) was read on an Epoch optical density plate reader by Biotek® following the manufacturer’s instructions. A dose-response curve was calculated using the standards provided by the Hygiena and the fumonisin parts per million (PPM) was calculated for each sample using the standard curve. As the distribution of fumonisin concentrations was positively skewed, the fumonisin concentrations were adjusted by transforming the scores to PPM^1/5^ to normalize scores prior to estimating correlations between fumonisin content and infected kernel counts. The data remained non-normal according to a Shapiro-Wilk normality test, but no better transformation could be determined. Because of this, two correlations were estimated: the first assumed normality and was between infected kernel counts and fumonisin PPM^1/5^ then again using averages per isolate. The second did not assume normality and used Spearman’s Rank Correlation Coefficient on both raw values and isolate averages. All correlations and scatterplots were presented using Excel or Matlab with a custom Python script.

Sequence Data Availability: All genome assemblies have been uploaded to NCBI under Bioproject ID number: PRJNA1047553.

## Supporting information

S1 Table Assembly statistics for all isolates used in this study. (XLSX)

S2 Table All chromosome names and the RefSeq ID. (XLSX)

S3 Table Average nucleotide identity scores. (XLSX)

S4 Table List of effectors differentially present or absent in the select most and least severe isolates used in Orthovenn3. Displayed are the chromosome, start codon position, Uniprot protein reference, and predicted protein product.
(XLSX)

S5 Table List of effectors that had a position change greater than 100kb between isolates FV54917 and FV-FL-03 and their BLAST function or conserved motif. *Secreted in xylem 14 had only an 84% identity with the *Fv* sequence but was included as it is well characterized in host-specific pathogenicity.
(XLSX)

S6 Table List of all “high effect” variants unique between FV54917 and FV-FL-03. Displayed for each isolate variant are the position of the variant (chromosome:nucleotide), what type of change was predicted, the gene type that was affected, and the gene reference number in the *Fusarium verticillioides* reference genome.
(XLSX)

S7 Table RagTag output alignment statistics for all *Fv* isolates aligned with the Fv7600 and Fv10027 reference genomes. (XLSX)

S8 Table Phenotypic data used in this research. (XLSX)

S1 Fig Phylogenetic tree produced from BUSCO gene alignment. *Fusarium circinatum* is rooted as the out-species. Branch lengths were standardized.
(TIF)

S2 Fig Phylogenetic tree produced from putative effectors as predicted by EffectorP. *F*. *circinatum* is rooted as the out-species. Branch lengths were standardized.
(TIF)

S3 Fig Average nucleotide identity heatmap for all *Fusarium* isolates. Red indicates a greater similarity while white is less similar.
(TIF)

S4 Fig Average nucleotide identity heatmap for only *Fusarium verticillioides* isolates. Two isolates (FV-FL-02 and FV32968) are also included. Red indicates a greater similarity while white is less similar.
(TIF)

S5 Fig Distribution of disease severity scores in all isolates. **A.** Histogram displaying the disease severity scores (average number of infected kernels) for individual isolates. **B.** Normal distribution graph of the disease severity scores.
(TIF)

S6 Fig Fumonisin *FUM* cluster and alignment and correlation. **A.** Heatmap of *FUM* biosynthetic gene cluster. Colors are based on orthologous alignment to the published gene sequences, darker brown is closely orthologous, blue indicates absence of the gene. The heatmap is arranged by isolate rows from lowest to highest similarity to the reference sequences. Gene names and UniProt protein numbers are on the x axis (“Gene.Uniprot”). **B.** Scatterplot between *FUM* cluster similarity and disease severity. Disease severity, as estimated by the average number of infected kernels, is on the X, and percent similarity of total *FUM* biosynthetic gene cluster per isolate are on the Y.
(TIF)

S7 Fig Circos plot between reference genomes Fv7600 and Fv10027_ITA. Reference Fv7600 is on the outside, Fv10027_ITA is the interior ring.
(TIF)

S8 Fig All predicted effectors. **A.** Location and density of all effectors predicted in all isolates. **B.** Heatmap showing total number of effectors for all isolates.
(TIF)

S9 Fig Distribution and density plots of variants. **A.** Variants of FV-FL-03. **B.** Variants of FV54917.
(TIF)

S10 Fig All predicted CAZyme classes’ orthogroups correlated with average isolate disease severity. **A.** Glycoside Hydrolase orthogroups. **B.** Carbohydrate esterase orthogroups. **C.** Auxiliary activities orthogroups. **D.** Carbohydrate binding molecule orthogroups. **E.** Polysaccharide lyase orthogroups. **F.** Glycosyltransferase orthogroups.
(TIF)

S11 Fig Boxplots representing two groups from what substrates the isolates were obtained. Groups are divided into “not maize” = blue boxplot and “maize” = orange boxplot. T-Test and Mann-Whitney p-values are displayed at the top. Disease severity (average number of infected kernels) is on the Y axis.
(TIF)
