# Supplementary figures and images for: Comparative genomics of Fusarium species causing Fusarium ear rot of maize
Source: PLoS One. 2024 Oct 18;19(10):e0306144. doi: 10.1371/journal.pone.0306144 (PMC11488721; doi:10.1371/journal.pone.0306144)

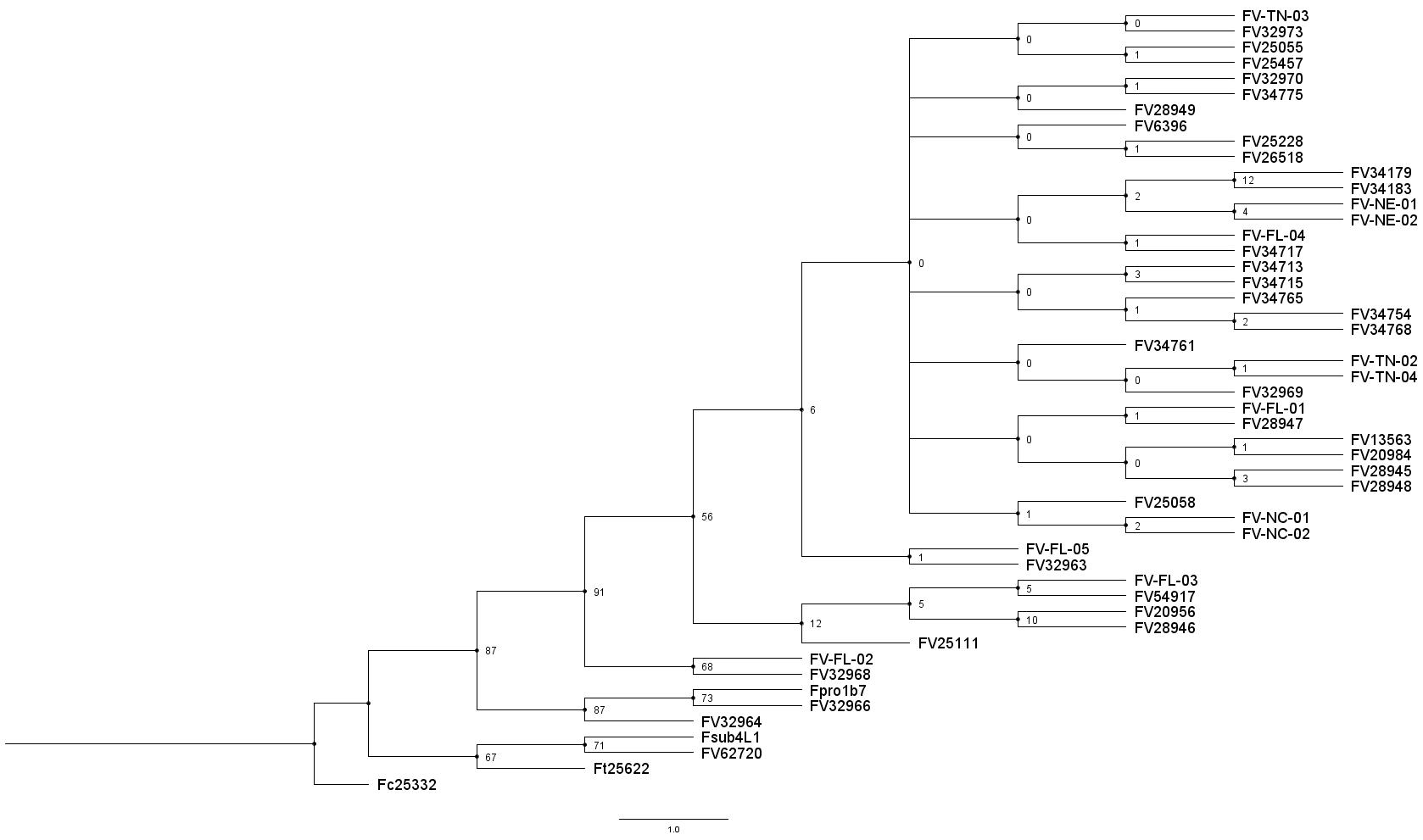

Supplement: S1 Fig — Fusarium circinatum is rooted as the out-species. Branch lengths were standardized. (TIF) [file pone.0306144.s009.tif]

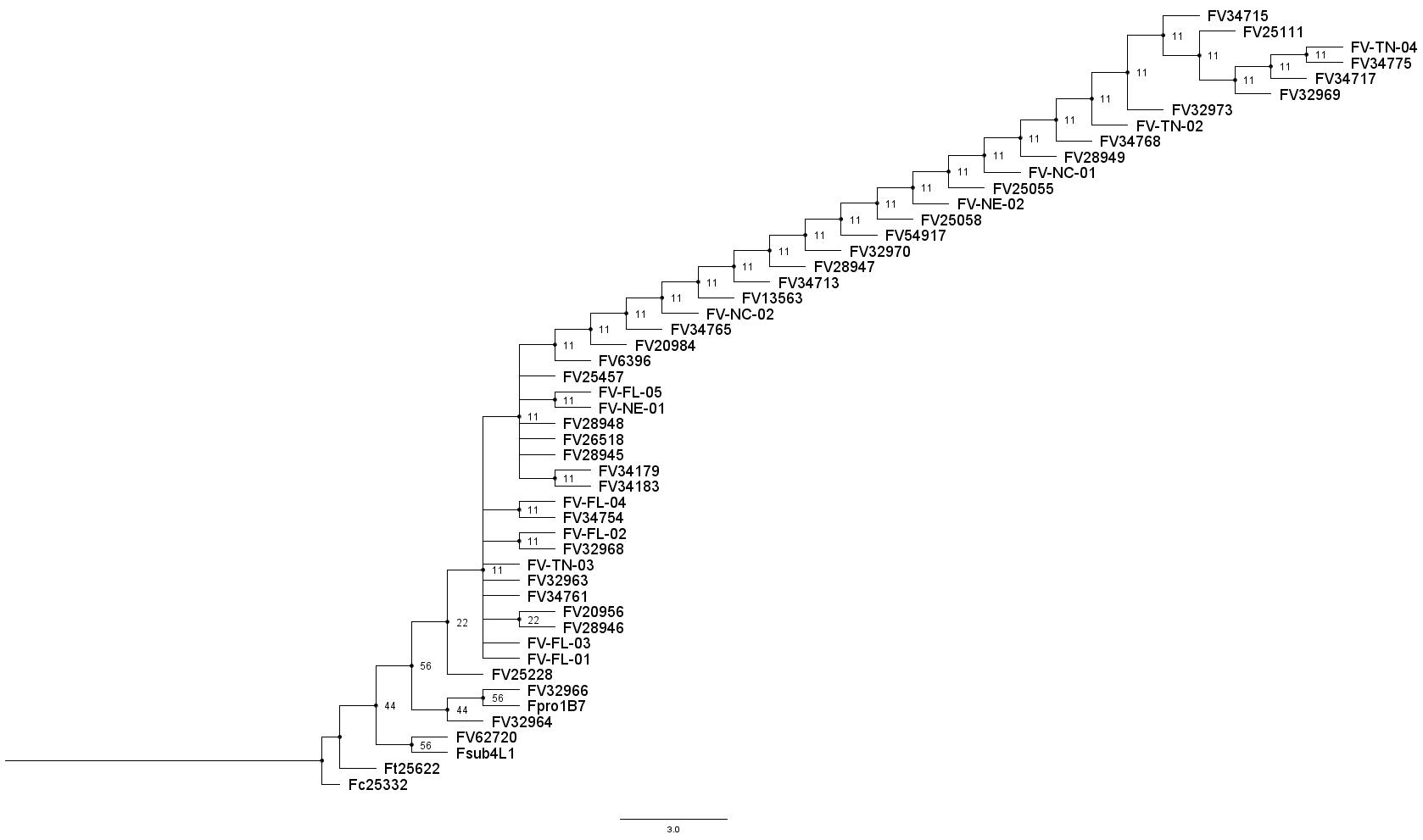

Supplement: S2 Fig — F. circinatum is rooted as the out-species. Branch lengths were standardized. (TIF) [file pone.0306144.s010.tif]

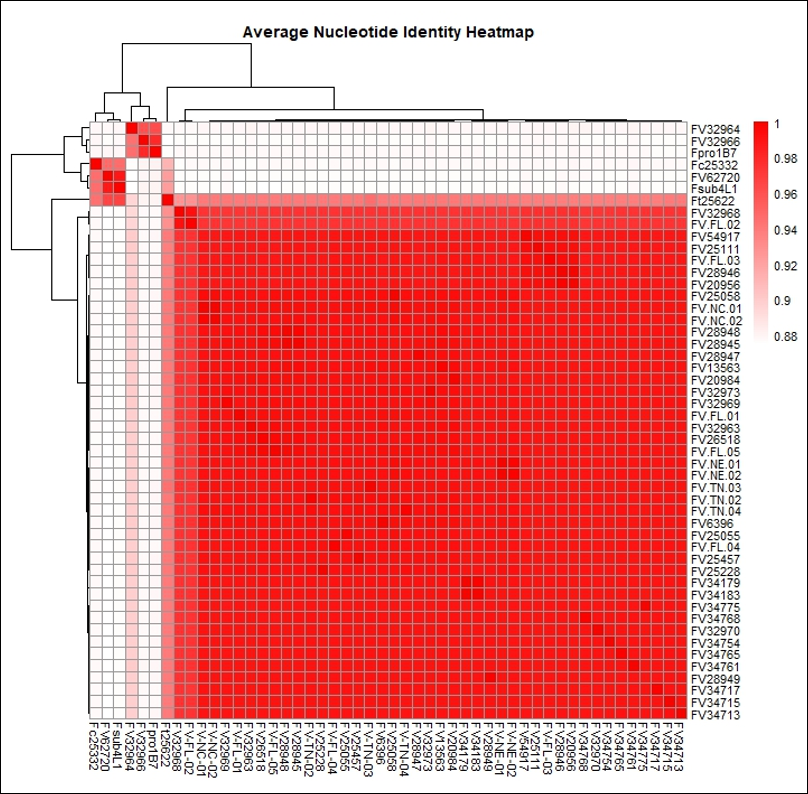

Supplement: S3 Fig — Red indicates a greater similarity while white is less similar. (TIF) [file pone.0306144.s011.tif]

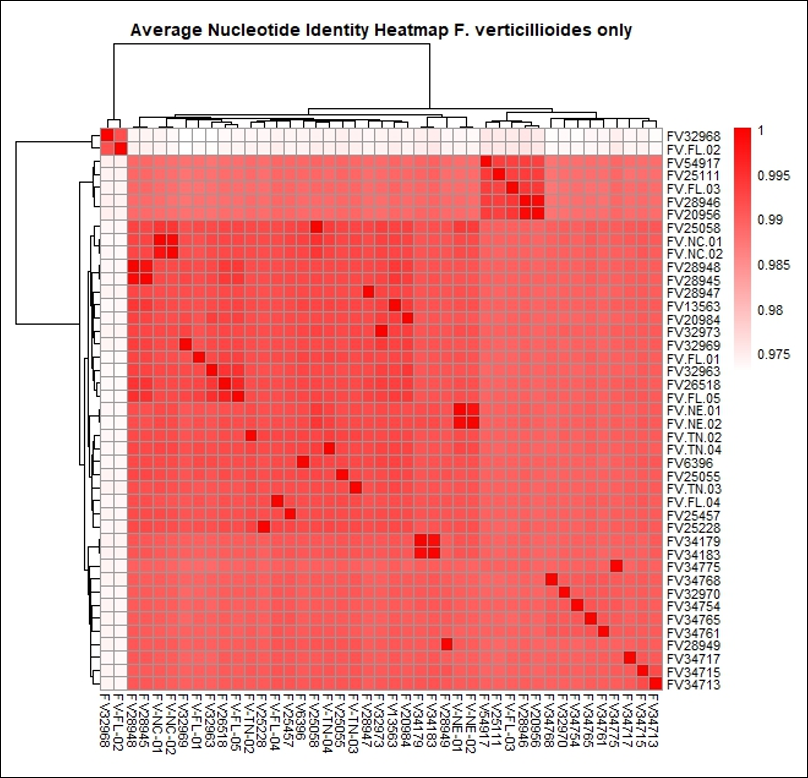

Supplement: S4 Fig — Two isolates (FV-FL-02 and FV32968) are also included. Red indicates a greater similarity while white is less similar. (TIF) [file pone.0306144.s012.tif]

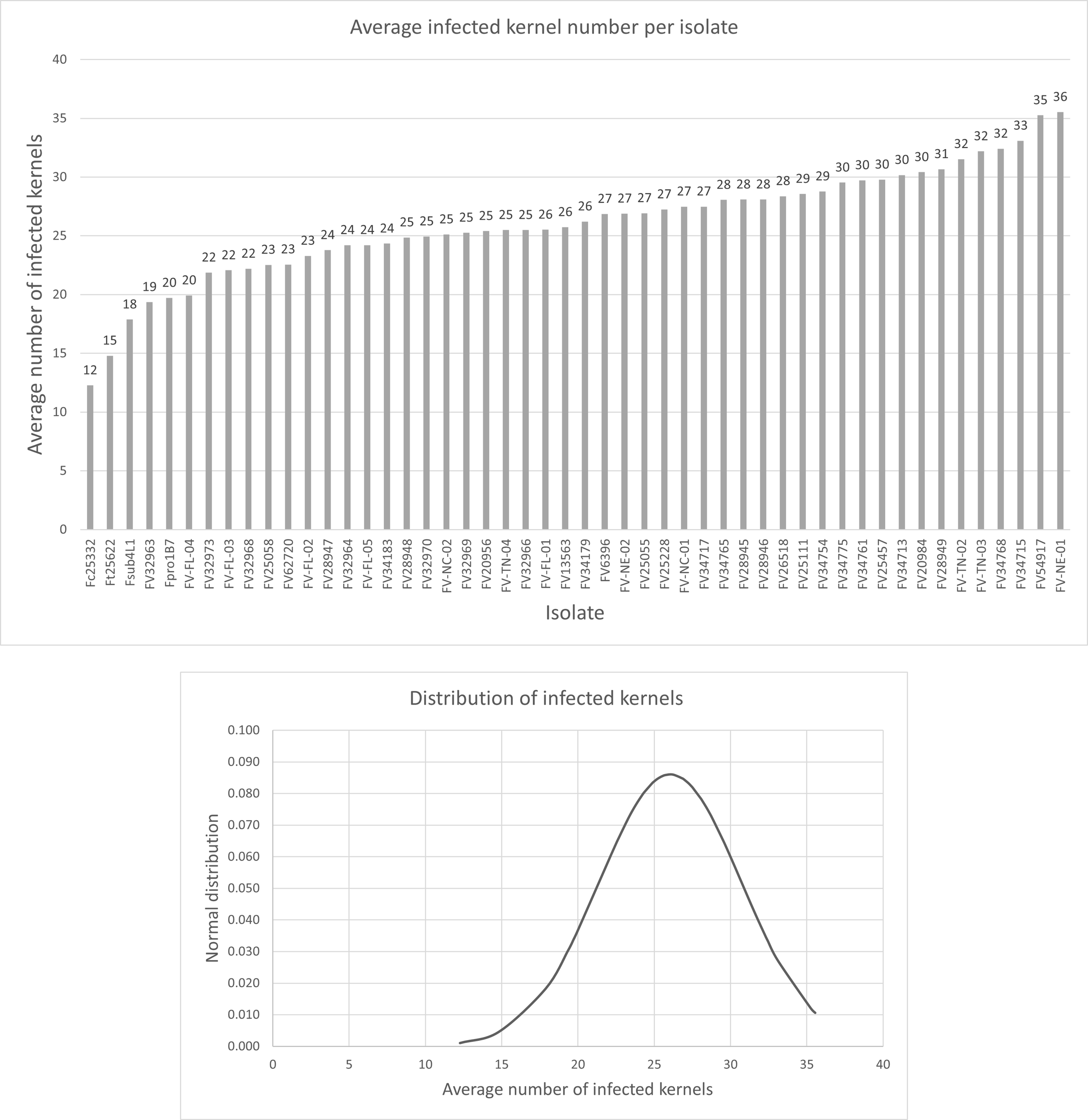

Supplement: S5 Fig — A. Histogram displaying the disease severity scores (average number of infected kernels) for individual isolates. B. Normal distribution graph of the disease severity scores. (TIF) [file pone.0306144.s013.tif]

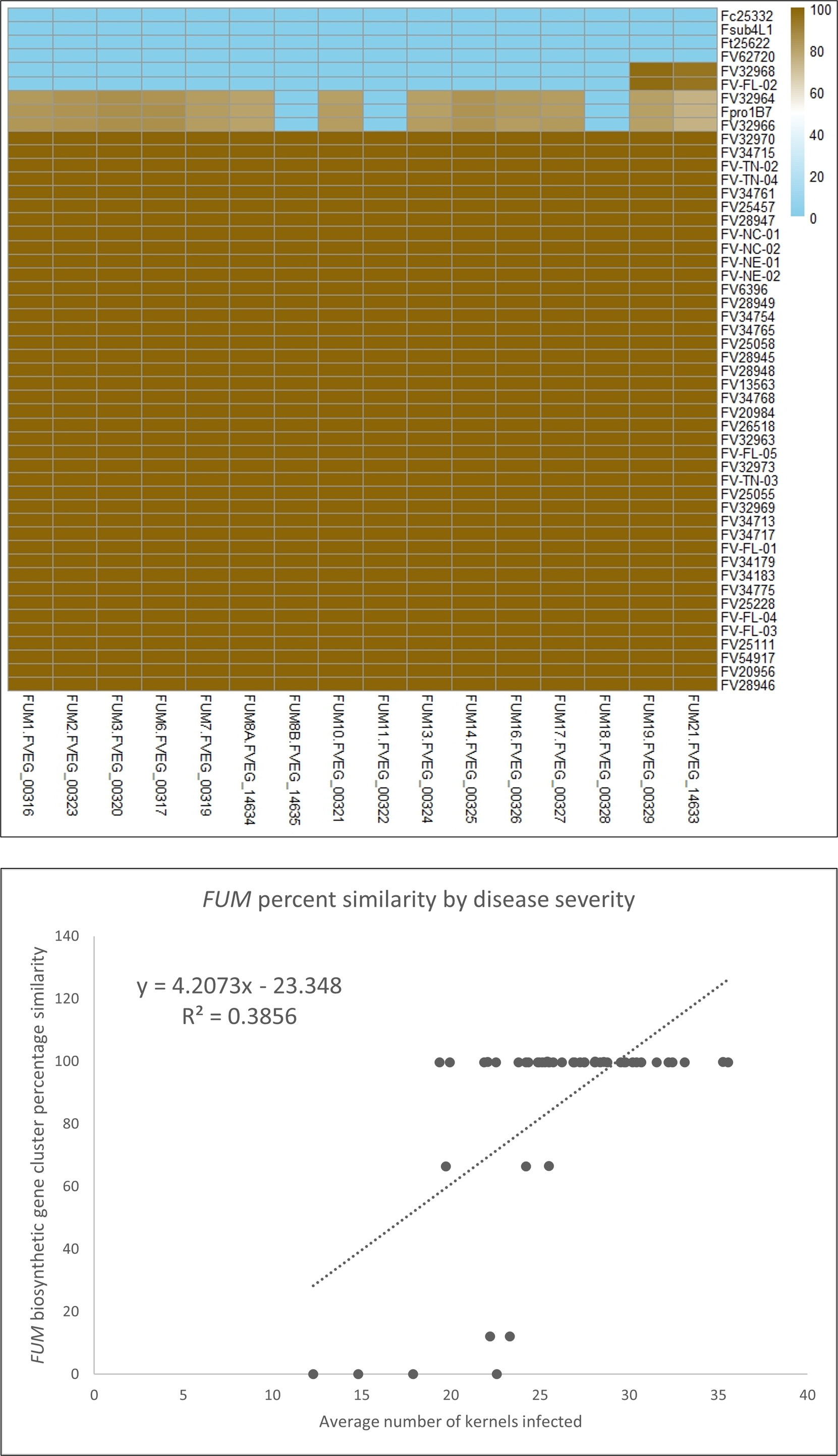

Supplement: S6 Fig — A. Heatmap of FUM biosynthetic gene cluster. Colors are based on orthologous alignment to the published gene sequences, darker brown is closely orthologous, blue indicates absence of the gene. The heatmap is arranged by isolate rows from lowest to highest similarity to the reference sequences. Gene names and UniProt protein numbers are on the x axis (“Gene.Uniprot”). B. Scatterplot between FUM cluster similarity and disease severity. Disease severity, as estimated by the average number of infected kernels, is on the X, and percent similarity of total FUM biosynthetic gene cluster per isolate are on the Y. (TIF) [file pone.0306144.s014.tif]

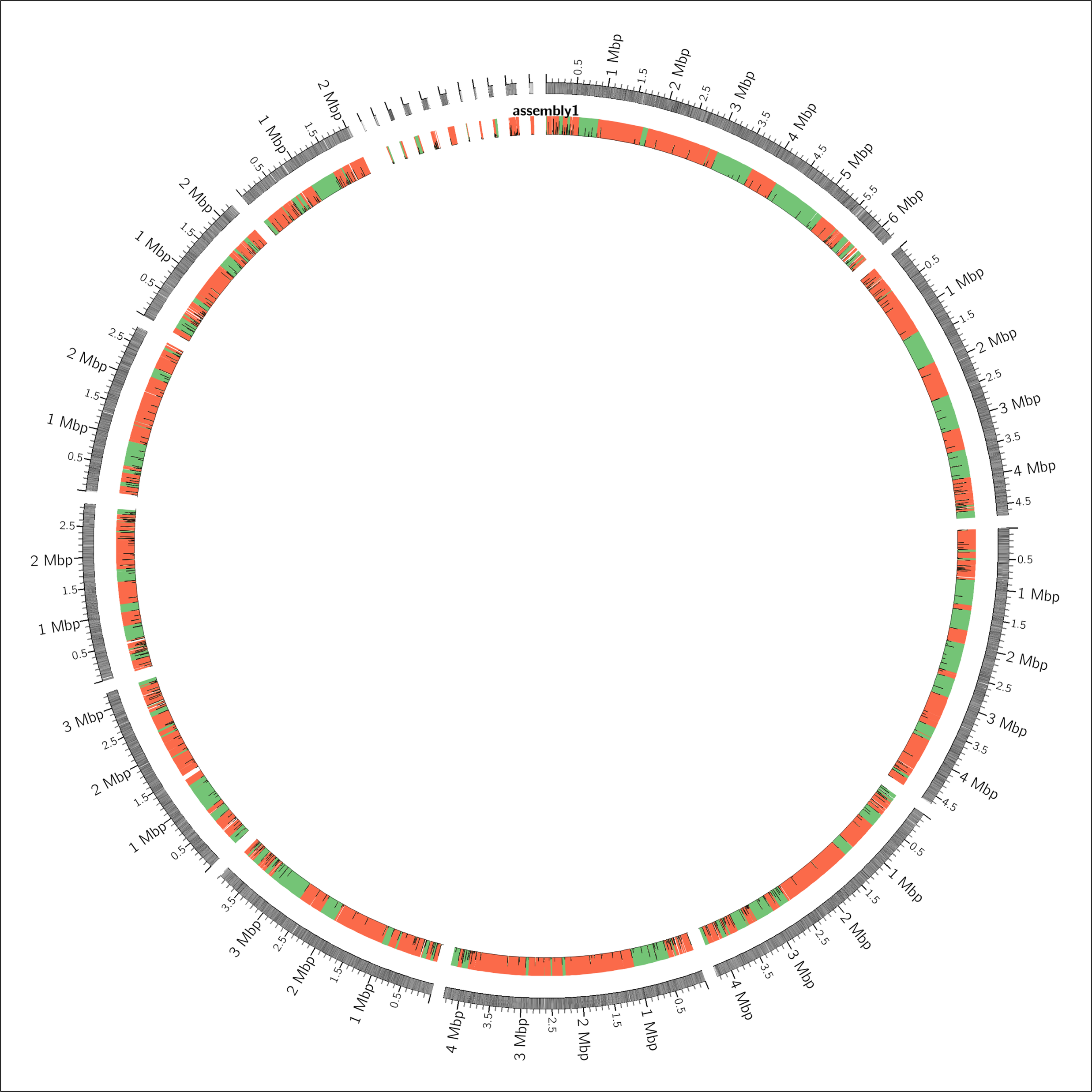

Supplement: S7 Fig — Reference Fv7600 is on the outside, Fv10027_ITA is the interior ring. (TIF) [file pone.0306144.s015.tif]

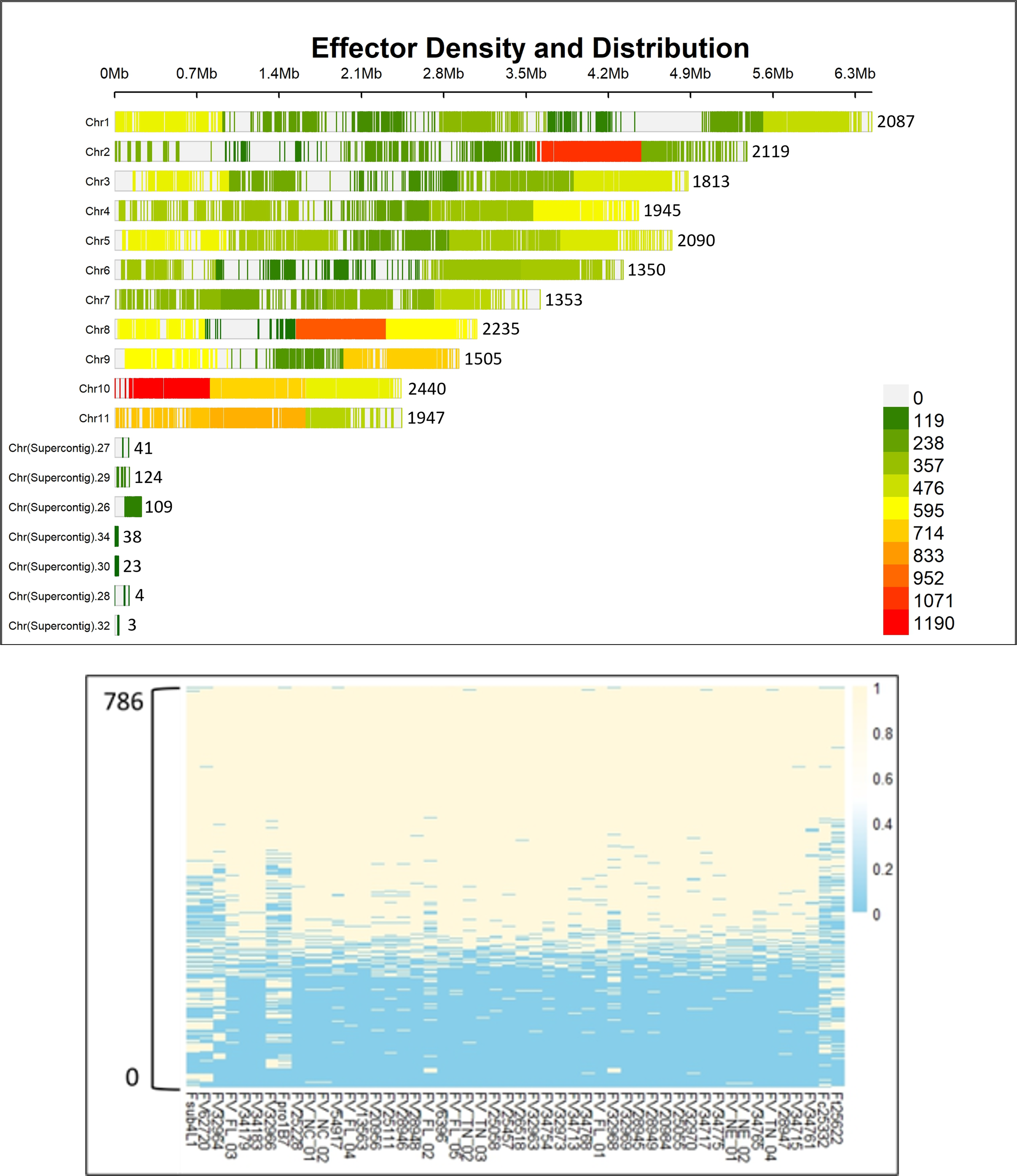

Supplement: S8 Fig — A. Location and density of all effectors predicted in all isolates. B. Heatmap showing total number of effectors for all isolates. (TIF) [file pone.0306144.s016.tif]

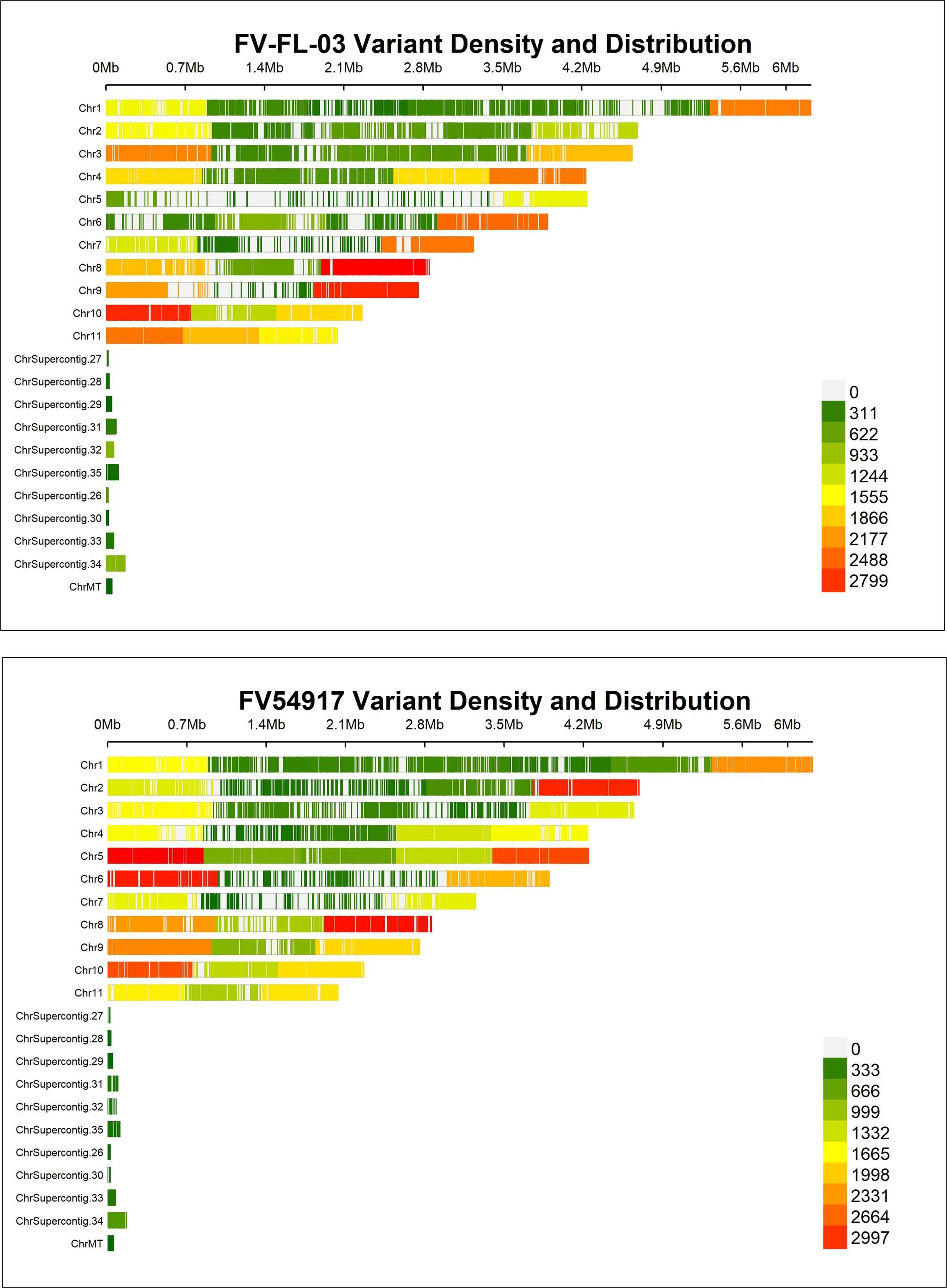

Supplement: S9 Fig — A. Variants of FV-FL-03. B. Variants of FV54917. (TIF) [file pone.0306144.s017.tif]

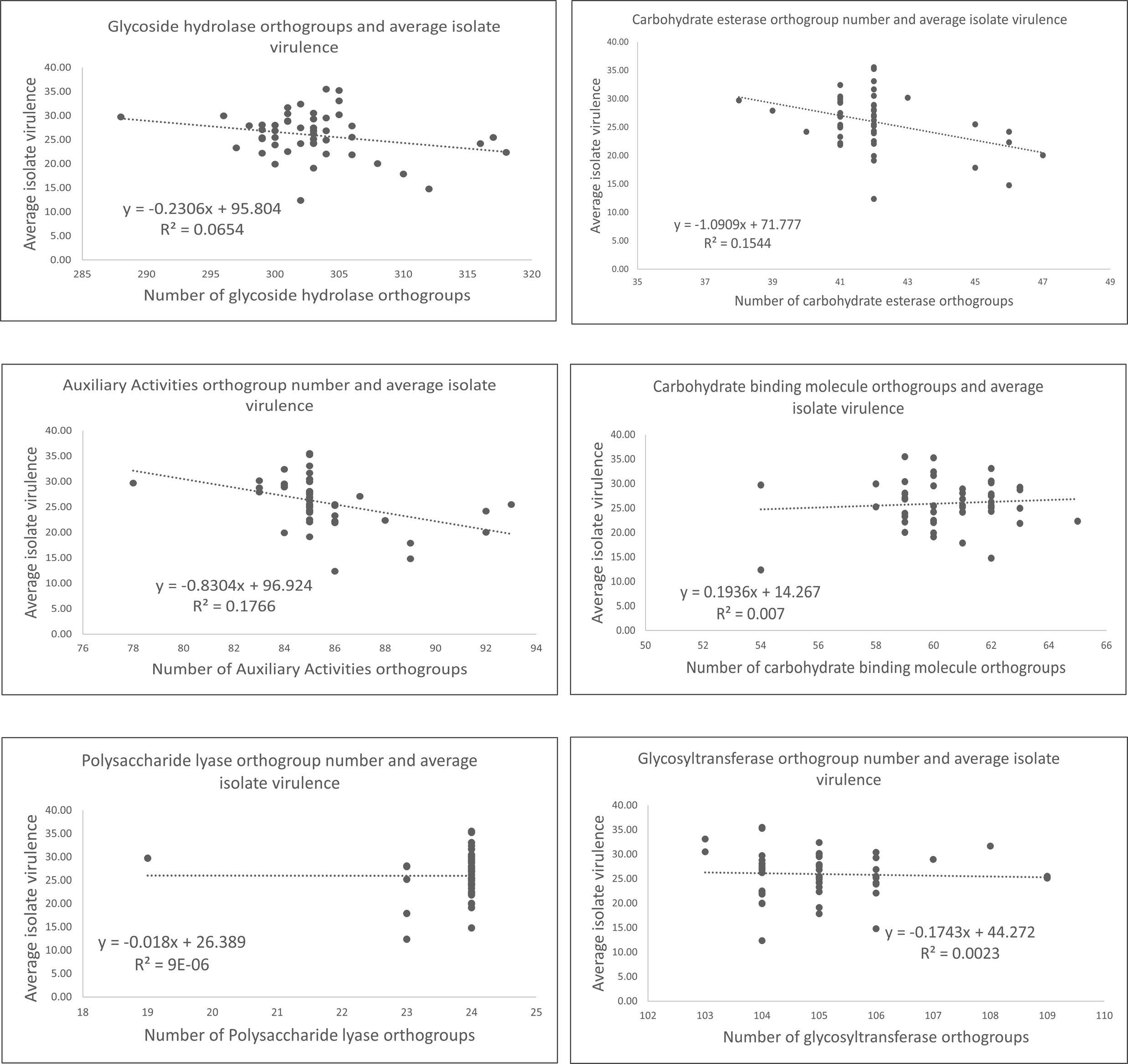

Supplement: S10 Fig — A. Glycoside Hydrolase orthogroups. B. Carbohydrate esterase orthogroups. C. Auxiliary activities orthogroups. D. Carbohydrate binding molecule orthogroups. E. Polysaccharide lyase orthogroups. F. Glycosyltransferase orthogroups. (TIF) [file pone.0306144.s018.tif]

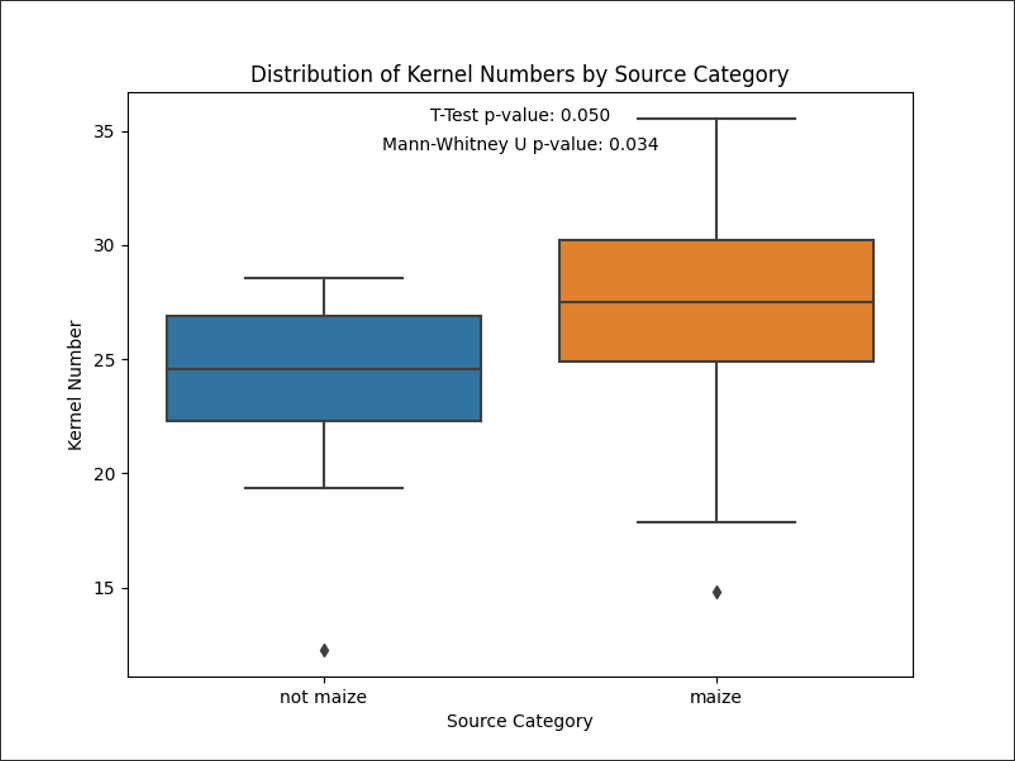

Supplement: S11 Fig — Groups are divided into “not maize” = blue boxplot and “maize” = orange boxplot. T-Test and Mann-Whitney p-values are displayed at the top. Disease severity (average number of infected kernels) is on the Y axis. (TIF) [file pone.0306144.s019.tif]
